# Repression and 3D-restructuring resolves regulatory conflicts in evolutionarily rearranged genomes

**DOI:** 10.1016/j.cell.2022.09.006

**Published:** 2022-09-29

**Authors:** Alessa R. Ringel, Quentin Szabo, Andrea M. Chiariello, Konrad Chudzik, Robert Schöpflin, Patricia Rothe, Alexandra L. Mattei, Tobias Zehnder, Dermot Harnett, Verena Laupert, Simona Bianco, Sara Hetzel, Juliane Glaser, Mai H.Q. Phan, Magdalena Schindler, Daniel M. Ibrahim, Christina Paliou, Andrea Esposito, Cesar A. Prada-Medina, Stefan A. Haas, Peter Giere, Martin Vingron, Lars Wittler, Alexander Meissner, Mario Nicodemi, Giacomo Cavalli, Frédéric Bantignies, Stefan Mundlos, Michael I. Robson

**Affiliations:** 1 Max Planck Institute for Molecular Genetics, Berlin, Germany; 2 Institute for Medical and Human Genetics, Charité Universitätsmedizin Berlin, Berlin, Germany; 3 Institute of Chemistry and Biochemistry, Freie Universität Berlin, Berlin, Germany; 4 Institute of Human Genetics, University of Montpellier, CNRS, Montpellier, France; 5 Dipartimento di Fisica, Università di Napoli Federico II and INFN Napoli, Complesso Universitario di Monte Sant'Angelo, Naples, Italy; 6 Department of Molecular and Cellular Biology, Harvard University, Cambridge, MA, USA; 7 Department of Stem Cell and Regenerative Biology, Harvard University, Cambridge, MA, USA; 8 Berlin Institute for Medical Systems Biology, Max Delbrück Center for Molecular Medicine, Berlin, Germany; 9 Charité-Universitätsmedizin Berlin, BCRT-Berlin Institute of Health Center for Regenerative Therapies, Berlin, Germany; 10 Centro Andaluz de Biología del Desarrollo (CABD), Consejo Superior de Investigaciones Científicas/Universidad Pablo de Olavide, Seville, Spain; 11 Kennedy Institute of Rheumatology, University of Oxford, Oxford, UK; 12 Museum für Naturkunde, Leibniz Institute for Evolution and Biodiversity Science, Berlin, Germany; 13 Broad Institute of MIT and Harvard, Cambridge, MA, USA; 14 Medical Research Council Human Genetics Unit, Institute of Genetics and Molecular Medicine, University of Edinburgh, Edinburgh, UK

**Keywords:** topologically associating domains, lamina-associated domain, enhancer-promoter specificity, DNA methylation, developmental gene regulation, evolution, loop extrusion, cohesin, CTCF, 3D genome organization

## Abstract

Regulatory landscapes drive complex developmental gene expression, but it remains unclear how their integrity is maintained when incorporating novel genes and functions during evolution. Here, we investigated how a placental mammal-specific gene, *Zfp42*, emerged in an ancient vertebrate topologically associated domain (TAD) without adopting or disrupting the conserved expression of its gene, *Fat1*. In ESCs, physical TAD partitioning separates *Zfp42* and *Fat1* with distinct local enhancers that drive their independent expression. This separation is driven by chromatin activity and not CTCF/cohesin. In contrast, in embryonic limbs, inactive *Zfp42* shares *Fat1*’s intact TAD without responding to active *Fat1* enhancers. However, neither *Fat1* enhancer-incompatibility nor nuclear envelope-attachment account for *Zfp42*’s unresponsiveness. Rather, *Zfp42*’s promoter is rendered inert to enhancers by context-dependent DNA methylation. Thus, diverse mechanisms enabled the integration of independent *Zfp42* regulation in the *Fat1* locus. Critically, such regulatory complexity appears common in evolution as, genome wide, most TADs contain multiple independently expressed genes.

## Highlights

• Novel genes can emerge in evolution without adopting or disrupting existing regulation
• TADs can be grossly restructured by chromatin activity independently of cohesin/CTCF
• NE attachment need not block gene activation or enhancer communication
• Context-dependent promoter silencing can refine enhancer usage in multi-gene TADs

## Graphical abstract

**Figure:**
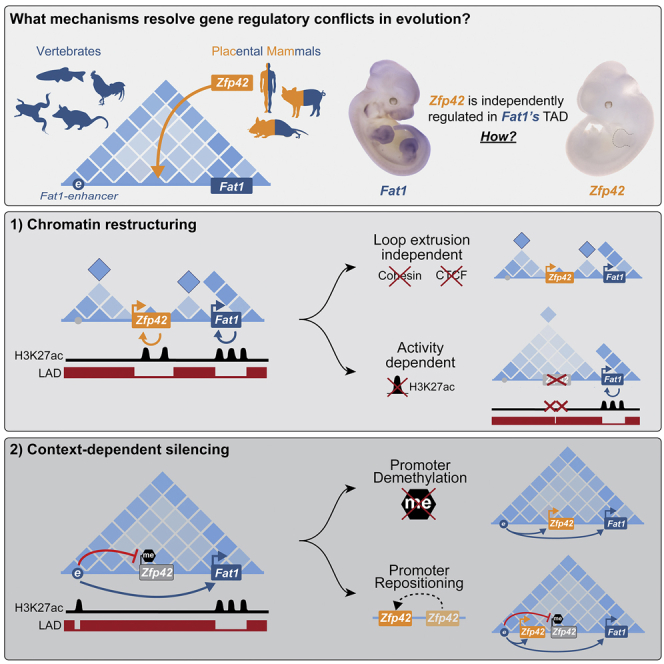

## Teaser

Multiple genetic and epigenetic mechanisms resolve the gene regulatory conflicts that inevitably arise during genome evolution.

## Introduction

During development, enhancers with diverse activities drive extraordinarily complex transcription at target genes in time and space (Long et al., 2016). Such enhancers can activate target genes often lying hundreds of kilobases away by physically contacting promoters in three-dimensional space via chromatin folding (Bonev and Cavalli, 2016; Furlong and Levine, 2018). This collectively allows many developmental loci to be regulated by complex modular ensembles of enhancers distributed within large gene regulatory landscapes (Robson et al., 2019). Modifying such regulatory landscapes and their transcriptional outputs is viewed as central for acquiring novel phenotypic traits in evolution (Wittkopp and Kalay, 2011). However, what mechanisms allow regulatory landscapes to be modified to incorporate novel activities without compromising their existing functions remains largely unknown.

In recent years, the 3D organization of the genome has emerged as one such modifiable feature that can alter a landscape’s activities. Regulatory landscapes are partitioned into preferentially self-interacting blocks termed topologically associated domains (TADs) by cohesin and the zinc-finger transcription factor CCCTC-binding factor (CTCF) (Dixon et al., 2016; Nora et al., 2012; Rao et al., 2014). Cohesin is thought to form TADs by progressively extruding chromatin loops until blocked by CTCF boundaries, thereby bringing distant loci into frequent spatial proximity (Fudenberg et al., 2016; Sanborn et al., 2015). In this way, TADs support gene regulation by continuously driving promoters to preferentially sample all enhancers within the same but not neighboring domains (Kane et al., 2021; Symmons et al., 2014; Zuin et al., 2022). As such, TADs and their enhancer landscapes are frequently conserved across cell types and species to sustain transcription in development and evolution (Dixon et al., 2012; Fraser et al., 2015; Harmston et al., 2017; Krefting et al., 2018). The importance of this general concept is demonstrated by TAD-disrupting genomic rearrangements that generate ectopic enhancer-promoter contacts driving gene misexpression and disease (Spielmann et al., 2018). However, in evolution, such re-wiring of enhancer-promoter interactions can also be a major source of phenotypic novelty (Acemel et al., 2017; Real et al., 2020). TADs thus provide a framework to understand the partitioning of regulatory information and how this can be modified in evolution to drive phenotypic innovation.

Nonetheless, this simple modular framework of interchangeable enhancers and promoters in shuffled TADs cannot alone explain how regulatory landscapes evolve. Although TADs transmit enhancer activities to all positions in a domain (Anderson et al., 2014; Zuin et al., 2022), newly emerged or reshuffled genes do not universally adopt all these regulatory inputs. Indeed, many TADs generated by evolution contain multiple genes with non-overlapping expression, despite all promoters contacting the same enhancers (Dixon et al., 2016). Likewise, mutations that create novel ectopic enhancer-promoter contacts within rearranged TADs frequently do so without driving corresponding gene misexpression or phenotypic change (Despang et al., 2019; Ghavi-Helm et al., 2019; Laugsch et al., 2019; Yin et al., 2021). Evolutionary altered regulatory landscapes must therefore employ additional mechanisms that further refine how and when promoters use enhancer activities. For example, strict enhancer-promoter compatibility or rendering promoters inert through repressive mechanisms like DNA methylation could allow modified landscapes to incorporate multiple divergently expressed genes (Furlong and Levine, 2018). Alternatively, isolation at the nuclear envelope (NE) in repressive lamina-associated domains (LADs) could sequester specific promoters away from enhancers within newly modified TADs (van Steensel and Belmont, 2017). However, the regulatory effects of LADs, enhancer-promoter compatibility, or DNA methylation are largely only inferred from correlative genome-wide studies or functional *in vitro* assays (Bergman et al., 2022; Borgel et al., 2010; Jagadeesh et al., 2021; Leemans et al., 2019; Zabidi et al., 2015). Consequently, it is unknown how extensively these features actually regulate endogenous genes in development and so can facilitate or constrain the evolution of regulatory landscapes.

Here, we address this by reconstructing how a new gene regulatory program could emerge during evolution within a more ancient TAD without disrupting its prior activities. By examining the *Zfp42*/*Fat1* locus, we find that a 300-kb region encompassing the *Zfp42* gene emerged within *Fat1*’s ancient TAD in placental mammals. We find two mechanisms that enabled independent *Zfp42* regulation while maintaining conserved *Fat1* expression. In embryonic stem cells (ESCs), the ancient TAD is partitioned to physically separate *Zfp42* and *Fat1* with distinct enhancers in smaller domains, thereby driving their independent activity. However, in embryonic limbs, *Zfp42* is rendered inert to *Fat1* enhancers that it contacts within the intact ancient TAD by highly context-dependent DNA methylation. Hence, multiple novel expression programs can be incorporated into a single locus during evolution through at least restructuring 3D-chromatin landscapes and selective promoter silencing. In this way, we demonstrate generalizable principles of how the genome resolves regulatory conflicts that inevitably arise in development and evolution.

## Results

### *Zfp42R* genes emerged within *Fat1*’s ancient TAD landscape

**Figure 1:**
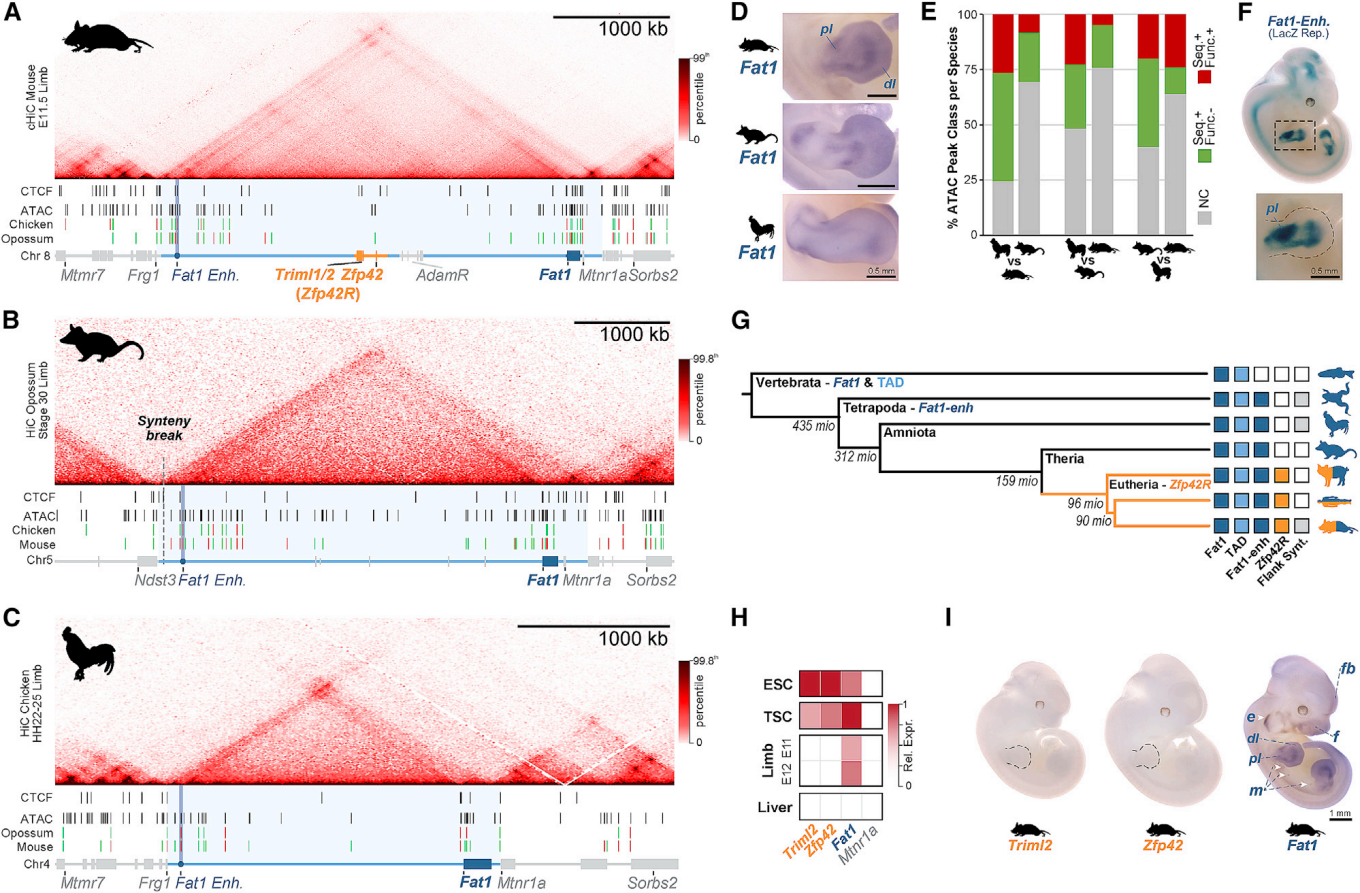
*Zfp42R* genes emerged with divergent expression in *Fat1*’s ancient TAD regulatory landscape (A–C) cHi-C or Hi-C from mouse (A), opossum (B), and chicken (C) embryonic limb buds with ATAC-seq and CTCF ChIP-seq peaks below. Genes are colored bars and lines indicate the TAD (light blue), the 293 kb sub-*Zfp42* region (*Zfp42R*, orange), and sub-Adam region (*AdamR*, gray). An ultra-conserved *Fat1* enhancer (*Fat1-enh*, blue circle) is also highlighted. ATAC peaks are colored by sequence conservation (seq) with or without matching functional ATAC signal (func.). Red (seq+, func.+); green (seq+, func.−); gray (seq−,func.−). (D) Species-specific *Fat1* WISH in embryonic limbs. n = 2–4. Scale bar, 0.5 mm. (E) Quantification of pairwise conservation of species ATAC-seq peaks. (F) LacZ reporter assay of mouse *Fat1-enh* in E11.5 embryos. n = 4 embryos. (G) Phylogenetic tree with presence of *Fat1*, the TAD, *Fat1-enh, Zfp42R*, or flanking synteny outside the TAD indicated. (H and I) Gene activity overview from Fantom5 CAGE expression (H) and WISH (I). *Fat1* WISH staining is seen in the ear (e), mammary glands (m), face (f), forebrain (fb), distal limb (dl), and proximal limb (pl). Trophoblast stem cells (TSCs). Scale bar, 1 mm. See Figures S1 and S2 and Tables S1, S2, and S6.

*Zfp42* (*Rex1*) is a well-studied pluripotency transcription factor that emerged from a retroposition duplication of *Yin Yang 1* (*Yy1*) in eutherian mammals (Kim et al., 2007; Masui et al., 2008). Capture Hi-C (cHi-C) in mouse E11.5 embryonic limbs revealed *Zfp42* locates in a ∼3.5 Mb CTCF-delimited TAD that contains eight genes (Figure 1A). Specifically, *Zfp42* is positioned within the TAD’s central 293-kb region (*Zfp42R*) together with two additional eutherian-specific genes *Triml1* and *Triml2* that are controlled by a bidirectional promoter (Figure S2D). Directly adjacent to *Zfp42R* lie *Adam26a*, 26*b*, and *34* (Adam region [*AdamR*]) which also arose from retroposition but specifically in rodents (Long et al., 2012). By contrast, *Fat1* and *Mtnr1a* are conserved across all vertebrates and position near the TAD’s telomeric boundary.

**Figure S1:**
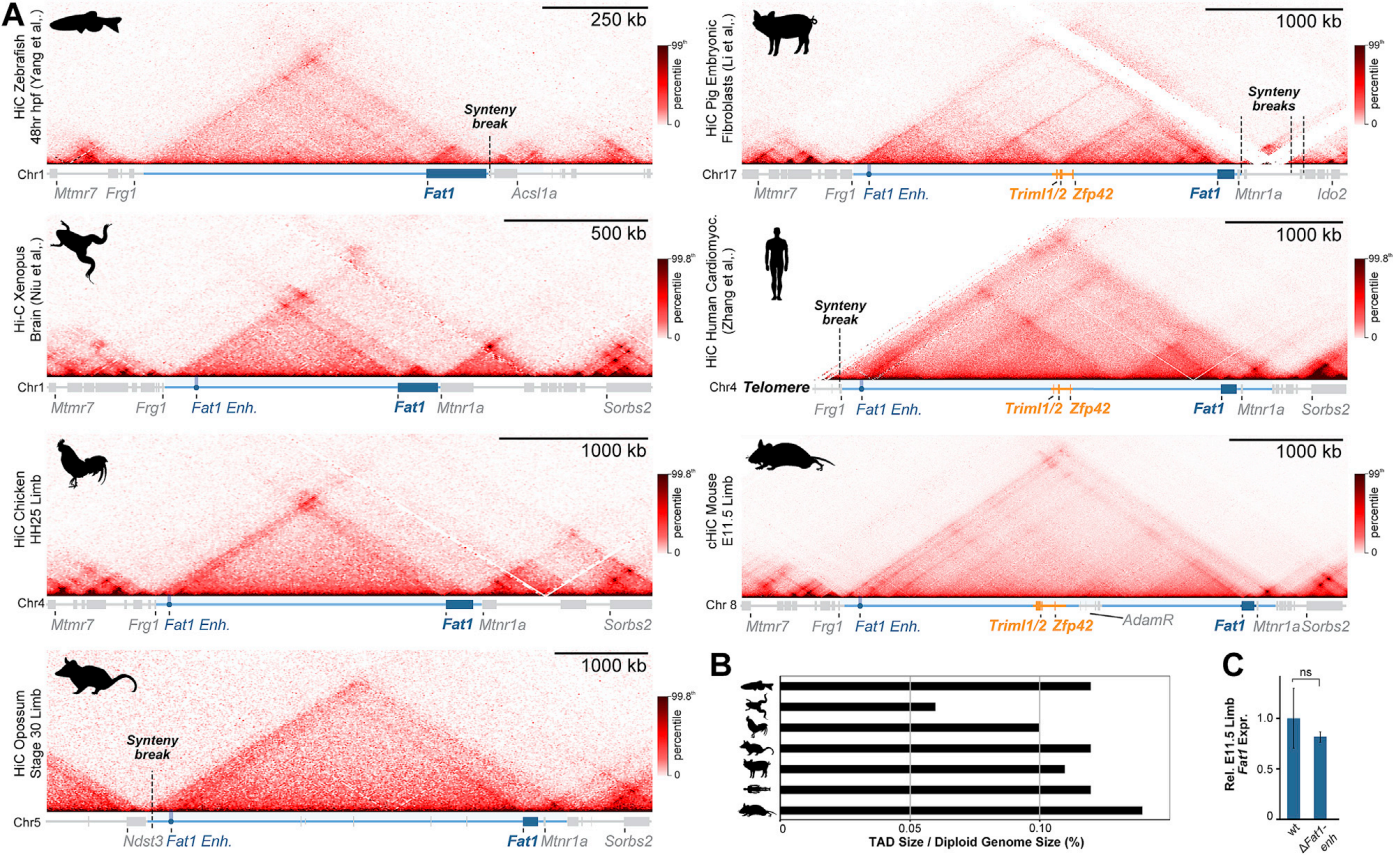
Extended TAD evolutionary analysis and impact of *Fat1-enh* deletion, related to Figure 1 (A and B) Hi-C from species spanning the vertebrate family tree (A) with quantification of TAD:Diploid genome size (B) (Li et al., 2020; Niu et al., 2021; Yang et al., 2020; Zhang et al., 2019). *Fat1* (dark blue box) has been universally maintained with a large gene desert and TAD (light blue line) whose size scales with diploid genome size. This is in spite of synteny breaks that relocate *Mtnr1a* (zebrafish), *Frg1* (opossum), *Mtmr7* (human), and *Sorbs2* (pig). The limb *Fat1-enh* emerged in tetrapods while *Mtnr1a* and its isolated TAD became incorporated into *Fat1*’s TAD in the Mammalia lineage. *Triml1*, *Triml2* and *Zfp42* emerged in eutherian placental mammals where they are universally conserved within the ancient TAD. Finally, retroposition events created a cluster of disintegrin metalloproteinases (*Adam26b*, *26a* and *34*) within the Adam gene cluster specifically in rodents (Brachvogel et al., 2002; Choi et al., 2004; Long et al., 2012). (C) RNA-seq expression effects of *Fat1-enh* deletion in E11.5 limbs. Error bars: standard deviation calculated from 4 biological replicates. non-significant (ns) p > 0.05.

We first tested how *Zfp42R* gene-emergence influenced the pre-existing regulatory landscapes of the ancient vertebrate *Fat1* and *Mtnr1a* genes. We thus applied Hi-C to morphologically stage-matched limb buds from opossum and chicken embryos and re-processed published Hi-C from tissues of diverse vertebrate species (Figure S1A; Li et al., 2020; Niu et al., 2021; Yang et al., 2020; Zhang et al., 2019). This revealed the placental-mammal TAD is conserved across vertebrates despite frequent flanking synteny breaks and has maintained a largely constant length relative to diploid genome size (Figures 1A–1C, S1A, and S1B; Jerković et al., 2017). However, only *Fat1* universally occupies the TAD in all tested vertebrate species with *Triml1/2* and *Zfp42* uniquely appearing in placental mammals (Figure S1A; Kim et al., 2007; Sadeqzadeh et al., 2014; Zhang et al., 2020). Similarly, *Mtnr1a* occupied a smaller isolated TAD in vertebrates and only became incorporated into *Fat1*’s TAD in the therian lineage (Figure S1A). Finally, the AdamR genes were most recently incorporated in rodents. Thus, *Fat1* and its conserved mono-gene TAD co-evolved in ancestral vertebrates prior to *Zfp42R* gene insertion in eutherians.

We therefore postulated the conserved TAD originally evolved to solely regulate *Fat1*. Supporting this, whole mount *in situ* hybridization (WISH) demonstrates that *Fat1* expression is conserved over ∼300 million years in mouse, opossum, and chicken embryonic limbs (Figure 1D; Helmbacher, 2018). Moreover, we find this preserved *Fat1* limb expression is driven by a conserved enhancer landscape. Specifically, matched limb assay for transposase-accessible chromatin using sequencing (ATAC-seq) identified 25–62 putative *cis*-regulatory elements per species which consistently cluster in the TAD’s distal arm or *Fat1*’s gene body (Figures 1A–1C). Of these, 5%–27% had conserved ATAC signal in pairwise species comparisons, and we tested one universally conserved distal element, *Fat1-enh*, in a mouse lacZ reporter assay (Figures 1E and 1F; Baranasic et al., 2021; see STAR Methods). Critically, *Fat1-enh* recapitulated a sub-set of *Fat1*’s overall expression in the proximal limb (pl) and neural tube, supporting the landscape's original function in regulating *Fat1* (Figure 1F). Moreover, deleting *Fat1-enh* had no effect on *Fat1* limb expression by RNA-seq, indicating multiple enhancers redundantly facilitate its expression (Figure S1C). Combined, this indicates *Fat1* co-evolved with a structurally stable TAD and functionally conserved enhancer landscape that drive its embryonic limb expression (Figure 1G). By contrast, *Zfp42R* genes emerged in the TAD in eutherian mammals without disrupting *Fat1*’s conserved expression.

### *Fat1* and *Zfp42R* genes are differentially expressed despite sharing a regulatory landscape

**Figure S2:**
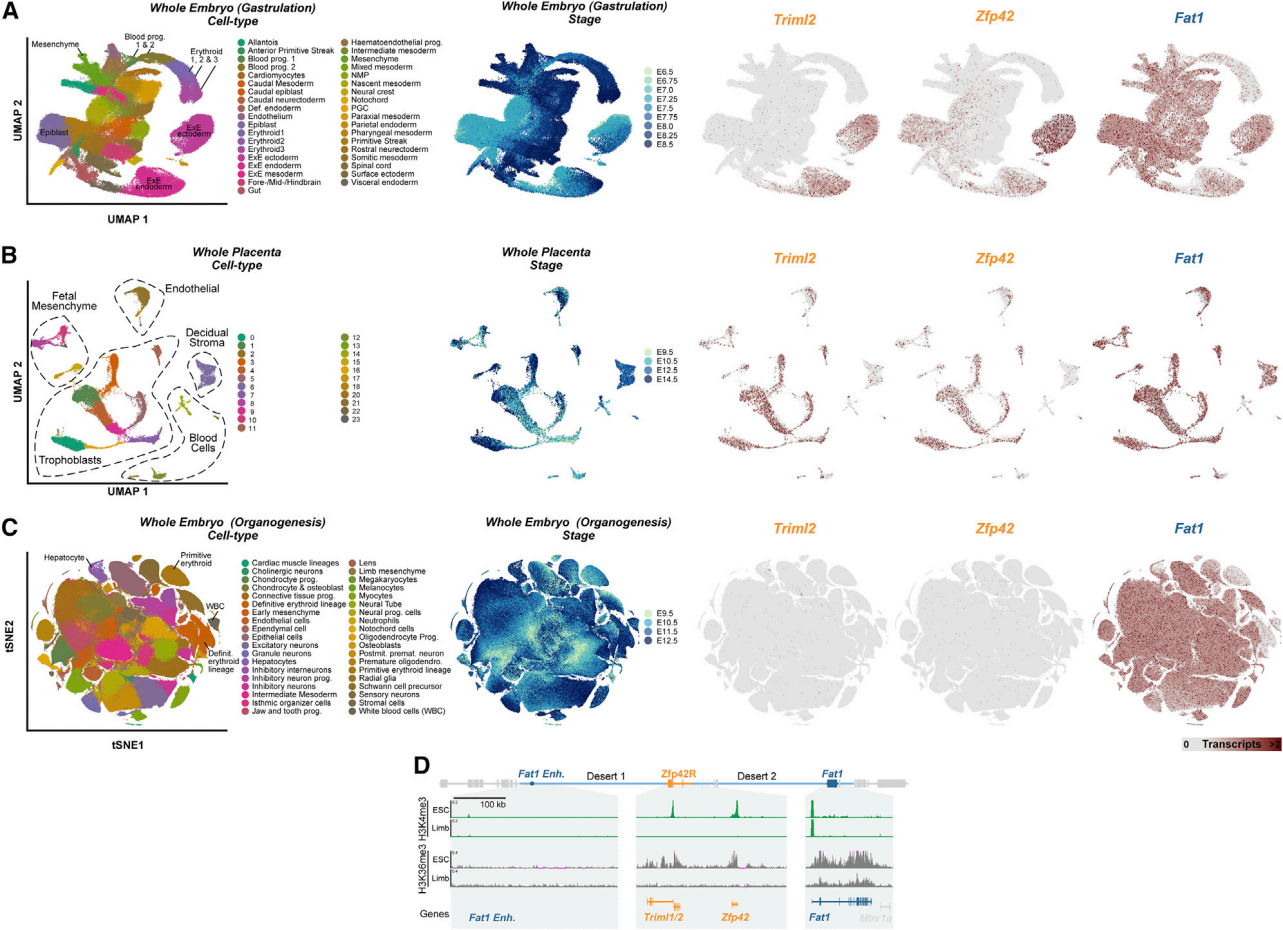
Extended *Zfp42R* and *Fat1* scRNA-seq gene expression analysis and expanded promoter mapping, related to Figure 1 (A–C) UMAPs from re-processed scRNA-seq from whole gastrulating embryos (A), the developing placenta (B), and whole embryos during organogenesis (C) (Cao et al., 2019; Marsh and Blelloch, 2020; Pijuan-Sala et al., 2019). UMAP embedding is colored according to cell type (left), developmental stage (middle), or expression of *Triml2, Zfp42* or *Fat1 (*right*)*. *Zfp42R* genes (*Triml2* and *Zfp42*) are expressed in the extraembryonic ectoderm and endoderm (A) and placental trophoblasts (B). *Zfp42* is also expressed in the E6.5 epiblast (A). *Fat1* is expressed widely in many tissues (A–C) but is absent, for example, in blood progenitors and erythroid cells (A and C). (D) Zoom of the centromeric TAD arm, *Zfp42R* and *Fat1* gene body with H3K4me3 and H3K36me3 ChIP-seq shown. Note that *Triml1* and *Triml2* are transcribed from a single shared bidirectional promoter as indicated by a single peak of H3K4me3 and broad H3K36me3 marking the transcribed gene body.

We now sought to determine how *Zfp42R* genes became functionally “wired into” *Fat1*’s pre-existing regulatory landscape. As TADs facilitate enhancer-promoter communication throughout evolutionarily and pathologically rearranged domains, we predicted *Zfp42R* genes would at least partially adopt *Fat1* expression (Real et al., 2020; Spielmann et al., 2018). However, gene expression profiling from available cap analysis of gene expression (CAGE) and single-cell RNA sequencing (scRNA-seq) atlases of mouse development revealed this is not the case (Figures 1H and S2A–S2C; Cao et al., 2019; FANTOM Consortium and the RIKEN PMI and CLST (DGT) et al., 2014; Lizio et al., 2015; Marsh and Blelloch, 2020; Pijuan-Sala et al., 2019). *Zfp42R* genes and *Fat1* are co-transcribed in ESCs, placental trophoblasts, and the extraembryonic ectoderm and endoderm (Figures 1H, S2A, and S2B; Masui et al., 2008; Zhang et al., 2020). Nevertheless, *Zfp42R* genes are inactive after gastrulation despite continued *Fat1* transcription in a variety of tissues, including E11 limb buds (Figures 1H and S2C). Confirming this, WISH demonstrated *Fat1* activity in the E11.5 limb, ear, snout, and mammary glands, whereas *Zfp42R* genes were undetectable, as previously reported (Figure 1I; Ciani et al., 2003; Helmbacher, 2018; Kim et al., 2011; Zhang et al., 2020). Thus, despite sharing a regulatory landscape, *Fat1* and *Zfp42R* genes are largely independently expressed. By contrast, *Mtnr1a* and *AdamR* gene expression was absent in all analyzed tissues, thereby excluding them from further analyses.

Collectively, this demonstrates that a monogenic TAD can incorporate new genes with independent expression patterns without disrupting its pre-existing gene’s expression. As such, currently unknown mechanisms must control which enhancers *Fat1* and *Zfp42R* genes utilize in their shared placental mammal landscape.

### *Fat1* and *Zfp42* independently utilize local enhancers in separated restructured domains in ESCs

**Figure 2:**
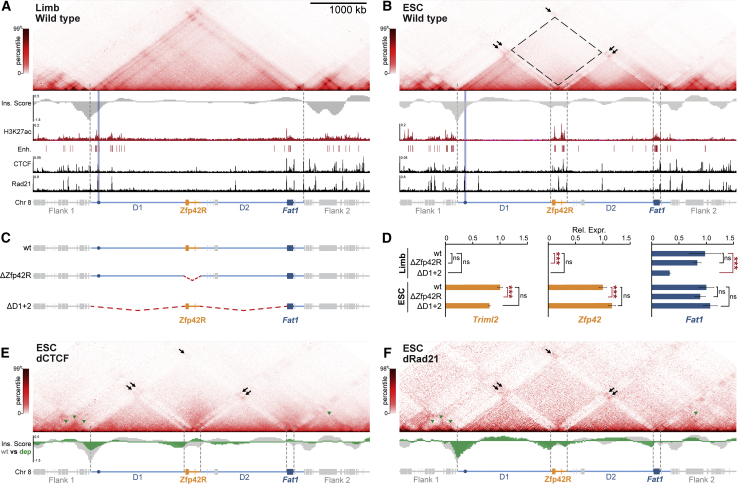
*Fat1 and Zfp42* independently utilize local enhancers in separated restructured domains in ESCs (A and B) cHi-C from E11.5 limb buds (A) and ESCs (B) with insulation score (Ins. Score), H3K27ac, CTCF & Rad21 ChIP-seq, and called putative enhancers below. For cHi-C, black arrows indicate interactions between active H3K27ac-marked regions and dotted rectangle indicates lost interactions between inactive D1 and D2. E11.5 limb cHi-C is reproduced from Figure 1. (C and D) Schematic of deletion mutants (C) with gene expression effects analyzed by RNA-seq (D). Error bars, SD calculated from 2–4 biological replicates per sample. ^∗∗∗^p < 0.001, ^∗^p < 0.05, non-significant (ns). (E and F) cHi-C from dCTCF (E) or dRad21 (F) ESCs with wild type (gray) or depletion (green) Ins. Scores below. Green arrows indicate flanking TADs disrupted by CTCF/Rad21 depletion. See Figure S3 and Tables S1, S2, and S4.

We therefore sought to identify the mechanisms adapting the ancient TAD landscape for independent *Fat1* and *Zfp42R* gene regulation in placental mammals. Thus, we mapped active enhancers and chromatin structure in mouse tissues where *Fat1* and *Zfp42R* genes are differentially expressed (E11.5 limbs) or active together (ESCs) (Figure 2). Significantly, both *Zfp42* and *Fat1* are dispensable for pluripotency and limb development, with the latter possessing functional redundancy with *Fat2*, *3*, or *4* (Ciani et al., 2003; Masui et al., 2008; Sadeqzadeh et al., 2014). As such, alterations to their regulation can be studied in ESCs and limbs without confounding effects.

**Figure S3:**
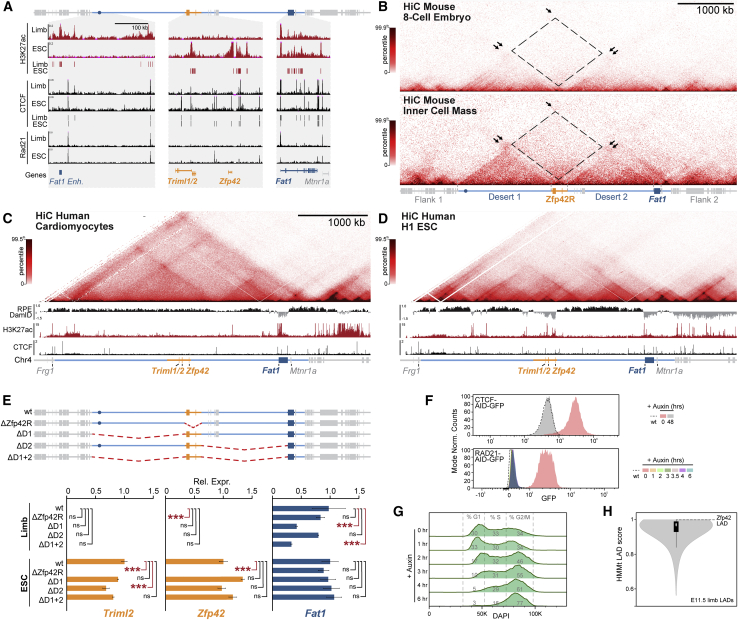
Confirmation of TAD disassembly in placental mammal pluripotency, CTCF/Rad21 depletion, and LAD signal strength, related to Figures 2 and 3 (A) Zooms of E11.5 limb and ESC H3K27ac, CTCF and RAD21 ChIP-seq with called enhancers or CTCF peaks below. (B) Low input Hi-C from mouse 8-cell embryos (top) and pluripotent cells from the inner cell mass (bottom) (Du et al., 2017). (C and D) Hi-C from human cardiomyocytes (C) and H1 ESCs (D) with corresponding H3K27ac, CTCF ChIP-seq and DamID shown below. Note DamID from retinal pigment epithelium (RPE) cells was used to define locus lamina-association when *Zfp42R* is inactive in differentiated cells. (E) Schematic of deletion mutants (top) with effects on gene expression determined by RNA-seq (bottom). Error bars: standard deviation calculated from 2–4 biological replicates per sample. ^∗∗∗^p < 0.001, ^∗^p < 0.05, non-significant (ns). (F) FACs distributions of GFP signal in CTCF-AID-GFP (top) and Rad21-AID-GFP (bottom) ESCs following indicated auxin treatments. (G) Distribution of cell-cycle phases in Rad21-AID-GFP ESCs showing rapid accumulation in S and G2/M within 6 h. To account for accumulation of Rad21-AID-GFP ESCs in G2/M phase caused by failed sister chromatid cohesion, cHi-C was performed on sorted G1 cells 3.5 h post-auxin addition (Liu et al., 2021). By contrast, due to technical difficulties plating fixed cells on coverslips, FISH was performed on unsorted 2 h-induced Rad21-AID-GFP ESCs where only moderate shifts in the G1:S:G2/M ratio were observed. (H) Genome-wide quantification of LAD scores from E11.5 limb DamID. The *Zfp42* LAD is highlighted and lies in the 88^th^ percentile of LADs genome wide.

In E11.5 limbs, ChIP-seq confirmed active H3K27ac-marked putative enhancers cluster near the TAD’s centromeric boundary and within *Fat1*’s gene body (Figures 2A and S3A; Andrey et al., 2017). However, in ESCs, a radically different TAD structure and underlying enhancer landscape emerged. Here, ESC-enhancer activities are redistributed into two distinct clusters found locally within *Zfp42R* and *Fat1*’s gene body (Figures 2B and S3A; Bauer et al., 2021). Correspondingly, *Zfp42R* and *Fat1* eliminate interactions with flanking gene deserts 1 and 2 (D1 & D2) and become insulated in individual active domains with these separate local enhancers. Combined, these alterations collectively partition the TAD into four domains (*D1*, *Zfp42R*, *D2*, and *Fat1*) in all tested cell types where *Fat1* and *Zfp42R* genes are active together, including 8-cell mouse embryos and human ESCs (Figures S3B–S3D; Du et al., 2017; Zhang et al., 2019). Thus, although evolutionarily stable, the ancient TAD has adopted a flexible structure in pluripotent eutherian cells that physically restricts *Fat1* and *Zfp42R* genes to contacting only their respective local enhancers.

We therefore tested if locus restructuring reflects which enhancers *Fat1* and *Zfp42R* genes utilize by generating a series of deletions in E11.5 embryos and ESCs (Kraft et al., 2015). Specifically, we eliminated the placental mammal-specific *Zfp42R* (ΔZfp42R) or the ancient D1 and D2 regions (ΔD1, ΔD2, or ΔD1+2) (Figures 2C and S3E). RNA-seq in mutant E11.5 limb buds revealed that *Fat1* expression was severely disrupted by deletion of the ancient D1 and D2 gene deserts but not the more recently emerged *Zfp42R*. Specifically, limb-wide *Fat1* expression was reduced by 56%–67% in ΔD1 and ΔD1+2 mutants, corresponding with the loss of putative centromeric limb enhancers that include the validated *Fat1-enh* (Figures 2D and S3E). By contrast, *Zfp42R* genes remained inactive in wild type and all mutant limbs. Hence, in later development, *Fat1* expression is driven by its ancient TAD regulatory landscape and distal enhancers, but these have no effect on *Zfp42R* gene expression.

In contrast, in ESCs, *Fat1* expression remained universally unaffected in ΔD1, ΔD2, ΔD1+2, and ΔZfp42R mutants (Figures 2C, 2D, and S3E). Similarly, *Zfp42R* genes were unaffected by single ΔD1/ΔD2 or combined ΔD1+2 deletions, except *Triml1/2* that showed mildly decreased activity in ΔD2 ESCs. Thus, in ESCs, *Fat1* and *Zfp42R* genes utilize only local enhancers for activity within their physically isolated domains in the dismembered TAD. As such, during pluripotency, *Fat1* and *Zfp42R* genes are functionally independent from one another in the now partitioned ancient regulatory landscape.

### The *Zfp42*/*Fat1* TAD is partitioned in ESCs independently of CTCF and cohesin

We next searched for the mechanism(s) that equip the ancient conserved TAD with such structural flexibility in ESCs. The current prevailing model is that TADs are formed by cohesin progressively extruding chromatin loops until blocked at CTCF boundaries (Fudenberg et al., 2016; Sanborn et al., 2015). As previously reported, binding sites for CTCF and the cohesin subunit Rad21 are enriched within *Zfp42R* specifically in ESCs (Figures 2B and S3A; Bonev et al., 2017). From this, we speculated that ESC-specific CTCF binding in *Zfp42R* blocks cohesin extrusion inside the center of the TAD, thereby driving locus restructuring.

We therefore globally depleted CTCF or Rad21 in ESCs (Figures S3F and S3G; Liu et al., 2021; Nora et al., 2017). As previously reported, most surrounding TADs and insulation collapsed once loop extrusion was either unconstrained (dCTCF) or eliminated entirely (dRad21) (Figures 2E and 2F; Liu et al., 2021; Nora et al., 2017; Rao et al., 2017). However, the *Zfp42/Fat1* locus surprisingly continued to partition into four discrete domains. Therefore, *Zfp42/Fat1* TAD partitioning in ESCs occurs independently of CTCF and loop extrusion and must instead be driven by one or several other dominant forces.

### Compartmentalization dominates in ESCs to partition the *Zfp42*/*Fat1* TAD

Beyond loop extrusion, chromatin is also antagonistically structured by the tendency of active or repressed chromatin to physically separate into mutually exclusive A and B compartments, respectively (Nuebler et al., 2018). Many B compartments then further interact with the NE to form repressive LADs (Falk et al., 2019; Rao et al., 2014; Robson et al., 2017). As the *Zfp42/Fat1* TAD restructures into active and inactive domains independently of cohesin, we reasoned that altered compartmentalization at the NE could drive its partitioning in ESCs.

**Figure 3:**
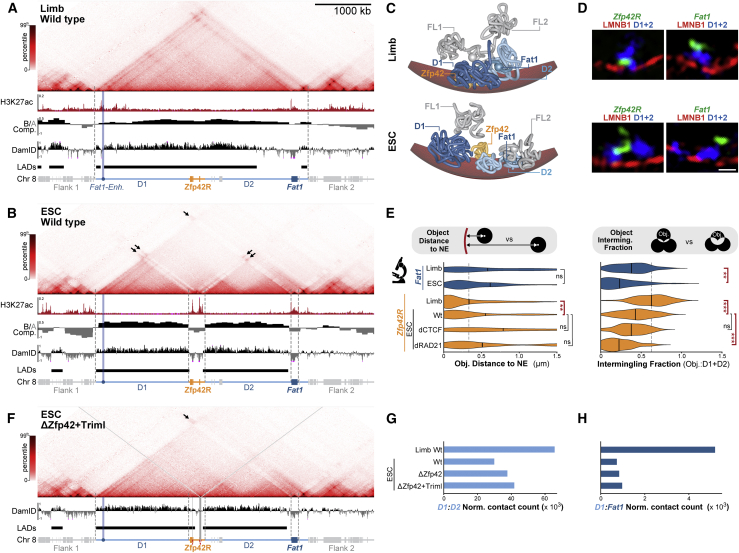
The *Zfp42/Fat1* TAD accommodates different chromatin environments in limb but is restructured into discrete compartments in ESCs (A and B) cHi-C from E11.5 limb buds (A) and ESCs (B) with H3K27ac-ChIP-seq, compartments, and Lamin B1 DamID tracks and called LADs below. cHi-C is reproduced from Figures 1 and 2. (C) Representative polymer model of locus with simulated NE (red) in E11.5 limbs (top) and ESCs (bottom). (D) Representative immunoFISH Z-slice with Lamin B1 (red), D1+D2 (blue) and *Zfp42R* or *Fat1* (green). Scale bar, 500 nm. (E) FISH measurements from wild-type limb or wild-type, CTCF-depleted (dCTCF), and Rad21-depleted (dRad21) ESCs. Object centroid distance to the NE (left) and intermingling fraction with D1+D2 (right) measurements are shown. Gray line highlights median limb values for reference. ^∗∗∗^p < 0.001, ∗∗p < 0.01, ^∗^p < 0.05, and non-significant (ns) from Welch's t test comparisons between indicated samples. n = 16–138 alleles of at least two biological replicates. (F) cHi-C and Lamin B1 DamID in Δ*Zfp42+Triml* ESCs with gray lines highlighting deleted H3K27ac regions. (G and H) Quantification of D1:D2 (G) and D1:*Fat1* (H) cHi-C interactions in indicated samples. See Figures S3 and S4 and Tables S1, S2, S3, and S5.

**Figure S4:**
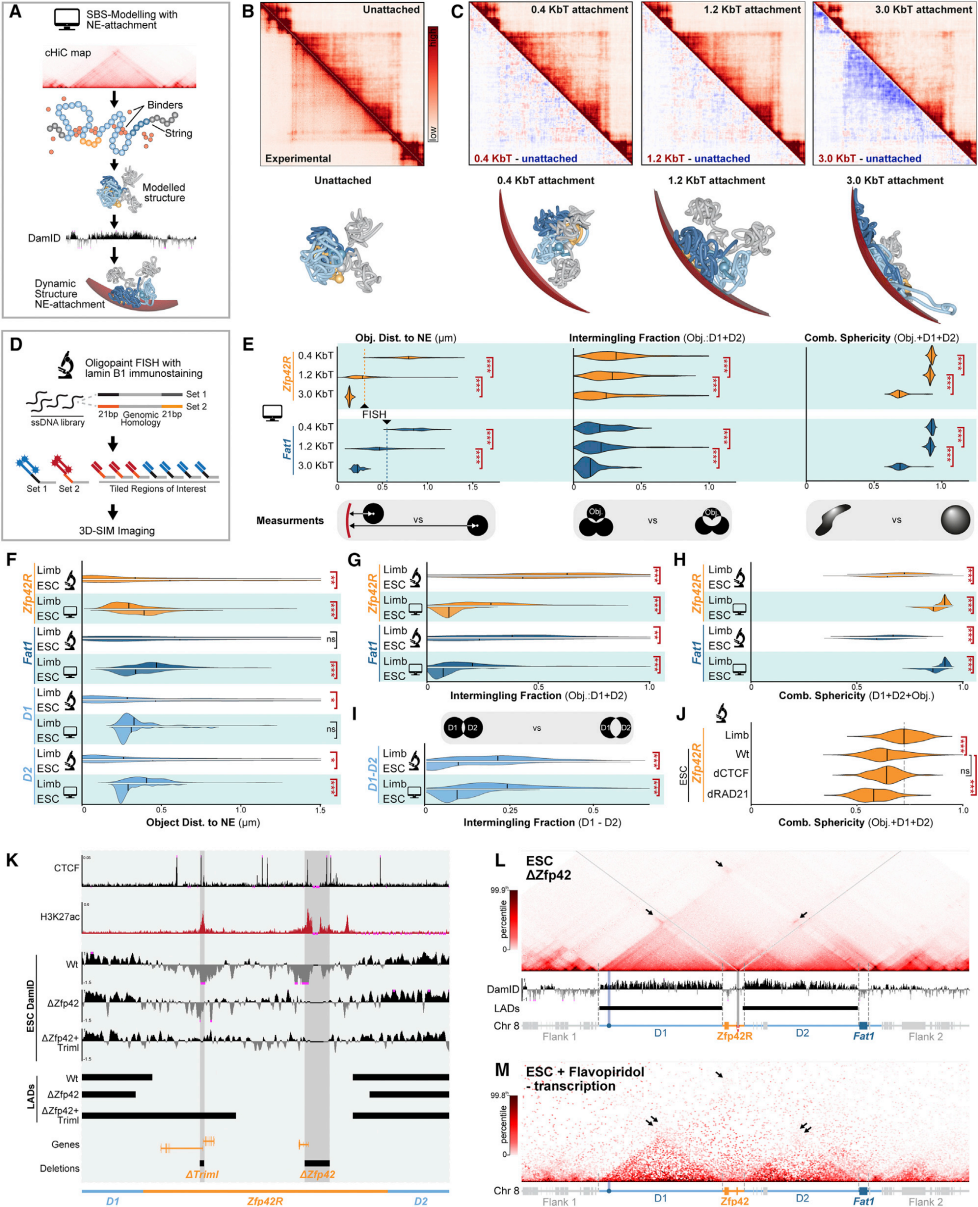
Comparison of SBS modeling with NE attachment and Oligopaint FISH and summary of ESC H3K27ac-deletion mutants, related to Figure 3 (A) Schematic representation of the modified strings-and-binders (SBS) polymer model. cHi-C contact maps were used to define PRISM-assigned chromatin binders. The chromatin polymer is then structured *in silco* through simulated DNA interactions created by the self-association between matching binders (Barbieri et al., 2012; Nicodemi and Prisco, 2009). Generated structures were subsequently dynamically attached to a modeled NE with polymer affinities determined from sample-matched DamID (see STAR Methods). (B and C) Reconstructed contact maps from simulated limb structures before (B) and after (C) NE attachment with 0.4, 1.2, and 3.0 kTb interaction energies. Corresponding subtraction maps and representative structures are shown below. n = 25–88 simulations. (D) Oligopaint FISH 3D-SIM imaging strategy. A library of single stranded DNA oligos with genomic homology and overhangs allow multiplexed staining of multiple regions of interest. (E) Quantification of object NE-distance (left), intermingling fraction (middle) and sphericity (right) for simulated limb structures following at indicated NE-attachment energies. 1.2 kTb was selected for further analysis as it produced NE-proximities without deforming the structure’s intermingling or sphericity relative to FISH measurements. (F–H) Comparison of simulated NE-attachment model at 1.2 kTb and experimental FISH data in wild-type E11.5 limbs and ESCs. Measurements are object NE-distance (F), intermingling fraction (G), and object sphericity with D1+D2 (H). (I) Comparison of simulated and observed D1 and D2 intermingling fraction. (J) Quantification of combined FISH sphericity of *Zfp42R* with D1+D2 in indicated samples. Gray line highlights median limb values for reference. ^∗∗∗^p < 0.001, ^∗∗^p < 0.01, and ^∗^p < 0.05 from Welch's t test comparisons. Non-significant (ns). FISH; n = 16–138 alleles of at least two biological replicates. (K) Zooms of *Zfp42R* with indicated ESC H3K27ac, CTCF ChIP-seq, Lamin B1 DamID tracks below. Shaded boxes highlight deleted H3K27ac regions. (L) cHi-C and DamID in Δ*Zfp42* ESCs. (M) Published Micro-C of JM8.N4 ESCs where transcription is inhibited by flavopiridol (Hsieh et al., 2020). Arrows indicate *Trim1/2*, *Zfp42* or *Fat1* interactions with active chromatin and evasion of heterochromatin.

To examine this possibility, we comprehensively mapped E11.5 limb and ESC compartments by Hi-C and corresponding NE attachment by DNA adenine methyltransferase identification sequencing (DamID-seq) (Figures 3A and 3B; Allou et al., 2021; Vogel et al., 2007). To further directly link altered 3D structure and NE attachment simultaneously at single loci, we additionally applied polymer modeling and 3D-structured illumination microscopy (3D-SIM) (see Figure S4 and STAR Methods for summary) (Barbieri et al., 2012; Nicodemi and Prisco, 2009) (Beliveau et al., 2015; Gustafsson et al., 2008; Schermelleh et al., 2008; Szabo et al., 2018, 2020). For the latter, chromatin was visualized through Oligopaint fluorescence *in situ* hybridization (FISH) and the NE through Lamin B1 immunolabeling (Figure S4D). Through this modeling and microscopy, we successfully measured simulated and observed structural features, including object NE-proximity, intermingling, and geometric shape (sphericity) (Figures 3E and S4F–S4J). In all cases, trends extracted from modeling and microscopy closely overlapped and so will be described below interchangeably. However, both measurements can be viewed together for comparison in Figures S4F–S4J.

This revealed that active and inactive chromatin is successfully combined in the intact TAD in limbs but is partitioned into discrete compartments in ESCs. Specifically, in limbs, the inactive *Zfp42R* is incorporated with D1 and D2 into a large NE-attached B compartment that spans most of the TAD, as reported in other differentiated mouse and human cell types (Figures 3A, S3C, and S3D) (Takebayashi et al., 2012; van Schaik et al., 2020; Zhang et al., 2019). The strength of this NE attachment is above average, lying in the 88^th^ percentile of LAD scores genome wide (Figure S3H). By contrast, *Fat1* locates within an active A compartment and, together with its limb *Fat1-enh*, remains locally detached from the NE in the same domain. Thus, the intact limb TAD simultaneously supports multiple interacting inactive and active compartments.

However, despite successfully mixing in limbs, active and inactive chromatin is partitioned at the locus in ESCs. Active *Fat1* and *Zfp42R* are now re-organized with their proximal enhancers into separated A compartments possessing reduced NE-proximity and lower intermingling with D1+D2 (Figures 3B–3E, S4F, and S4G). Conversely, D1 and D2 themselves remain as NE-attached B compartments but now poorly intermingle together (Figures 3B, S4F, and S4I). As a result, collective *Zfp42R+D1+D2* or *Fat1+D1+D2* sphericity is reduced in ESCs, thereby indicating the objects now exist as separated structures in a non-spherical elongated state (Figures 3B–3D and S4H). Critically, this partitioning in compartments further intensifies when loop extrusion is eliminated, as exemplified by reduced *Zfp42R* intermingling and combined sphericity with D1+D2 in dRad21 ESCs (Figures 3E and S4J). Combined, this suggests that antagonistic compartmentalization defined by chromatin state overrides loop extrusion in ESCs to disassemble the TAD.

To test this, we removed the active epigenetic signature from *Zfp42R* that is suggested to drive A compartments to physically separate from inactive chromatin (Rosencrance et al., 2020; Xie et al., 2022). Deleting the H3K27ac peaks that reportedly represent the *Zfp42* and *Triml1/2* promoters, and most nearby enhancers collapses TAD partitioning in ESCs (Sima et al., 2019). Specifically, removing H3K27ac at *Zfp42* (ΔZfp42) and then *Triml1/2* (ΔZfp42+Triml) causes NE attachment to progressively invade *Zfp42R* in ESCs (Figures 3F, S4K, and S4L). In parallel, D1+D2 cHi-C interactions are also progressively restored with both one another and *Zfp42R*, thereby partially reassembling the TAD (Figure 3G). However, full TAD restoration is prevented by *Fat1*’s continued association in an isolated active compartment that maintains its independent separation (Figure 3H). Critically, similar collapsed partitioning is not observed following ablation of transcription alone, confirming mutants disrupt locus structure by eliminating active chromatin and not *Zfp42R* gene transcription per se (Figure S4M; Hsieh et al., 2020).

In summary, chromatin state dominates the locus’s structure in ESCs through compartmentalization, thereby isolating *Fat1* and *Zfp42R* genes with independent enhancers. During pluripotency, *Zfp42R* and *Fat1* can thus operate as independent entities in their shared regulatory landscape.

### LADs neither directly silence nor indirectly insulate *Zfp42R* genes

We now sought to dissect the later embryonic limb situation where *Zfp42R* genes remain inactive, despite contacting *Fat1* and its distal limb (dl) enhancers in a shared intact TAD. LADs are compacted heterochromatin domains known to repress transcription (Leemans et al., 2019; Ou et al., 2017; Robson et al., 2016). Accordingly, we reasoned that the LAD environment of *Zfp42R* inactivates its genes in limbs, either by direct repression or by indirectly blocking communication with *Fat1* enhancers.

**Figure 4:**
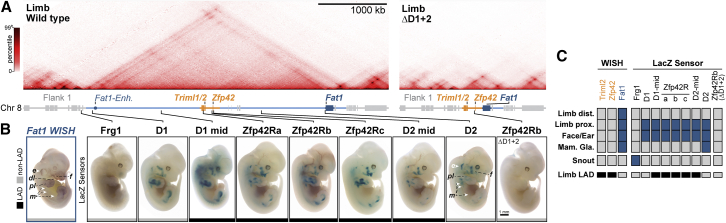
NE attachment neither blocks *Zfp42R* gene activation nor their communication with *Fat1* enhancers (A) Hi-C from wild-type and ΔD1+2 E11.5 limb buds, with the former reproduced from Figure 1. (B) Staining of endogenous *Fat1* (WISH, *left*) or integrated *β-globin* LacZ sensors (LacZ, *right*) in E12.5 embryos. n = 4–10 embryos. Integration sites are indicated by lines and their NE attachment in limb by black (LAD) or gray (non-LAD) boxes. Staining is indicated in the ear (e), distal limb (dl), proximal limb (pl), mammary glands (m), and face (f). Scale bar, 1 mm. (C) Summary of gene, enhancer, and sensor activities with LAD-status indicated. See Figure S5 and Tables S1, S2, and S3.

**Figure S5:**
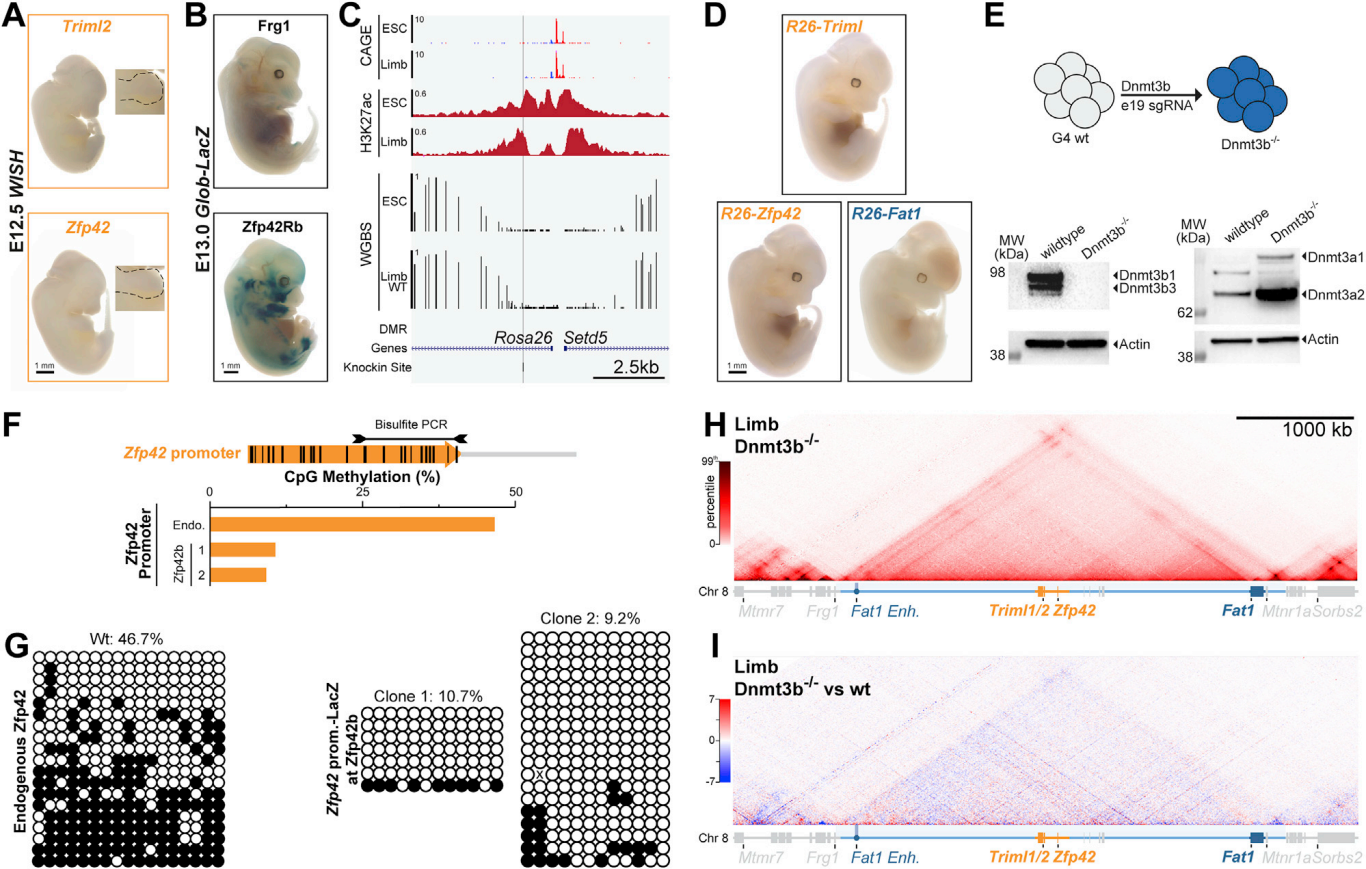
Testing intrinsic promoter activities, bisulfite conversion cloning and generation and analysis of DNMT3A/B knockouts, related to Figures 4 and 5 (A) *Triml2* and *Zfp42* WISH stainings in E12.5 embryos. Scale bar is 1 mm. (B) Staining from *β-globin* LacZ sensors integrated at *Frg1* and *Zfp42Rb* in E13.0 embryos. n = 4–10 stained embryos per position. (C) Zoom of *Rosa26* safe harbor locus with CAGE, H3K27ac ChIP-seq and WGBS shown. Sensor integration site is indicated by the gray bar with insert transcription orientation matching *Rosa26*. (D) LacZ stainings from E12.5 embryos with sensors driven by indicated promoters integrated at the *Rosa26* locus. (E) Strategy for *Dnmt3b* knockout in ESC clones with western blot confirmation shown below. DNMT3A increases following loss of DNMT3B. (F) Schematic of bisulfite conversion cloning strategy with quantification of methylated CpGs at the endogenous or transplanted *Zfp42* promoter in E11.5 limbs. (G) Corresponding lollipop diagrams of *Zfp42* promoter methylation (black methylated and white unmethylated CpGs). (H and I) cHi-C from E11.5 DNMT3B^−/−^ limb buds (H) with subtraction to wild type shown below (I).

To investigate this, we mapped the availability of *Fat1* regulatory activity by integrating minimal β-globin (*Glob*) promoter-LacZ sensor constructs at seven positions within and one position outside the TAD (Symmons et al., 2014). LacZ staining of E12.5 embryos revealed all seven intra-TAD “sensor” locations recapitulated most of *Fat1*’s expression pattern, although subtle positional differences were observed (Figures 4A–4C). Critically, this sensor activity was abolished when integrated at Zfp42Rb in ΔD1+2 embryos that lack most of the TAD, confirming its dependence on *Fat1* enhancers (Figures 4A–4C). Likewise, the sensor was activated entirely distinctly from *Fat1* in the snout and external genitalia when insulated from its enhancers following integration outside the TAD near *Frg1* (Figures 4B and S5B). Thus, the genomic positions of the inactive *Zfp42R* genes can paradoxically sample *Fat1* enhancers within the TAD despite extensive surrounding and intervening heterochromatic LADs. In short, LADs neither directly silence *Zfp42R* genes nor indirectly block their communication with *Fat1* enhancers.

### Strict enhancer-promoter specificity cannot account for *Zfp42R* gene inactivity

**Figure 5:**
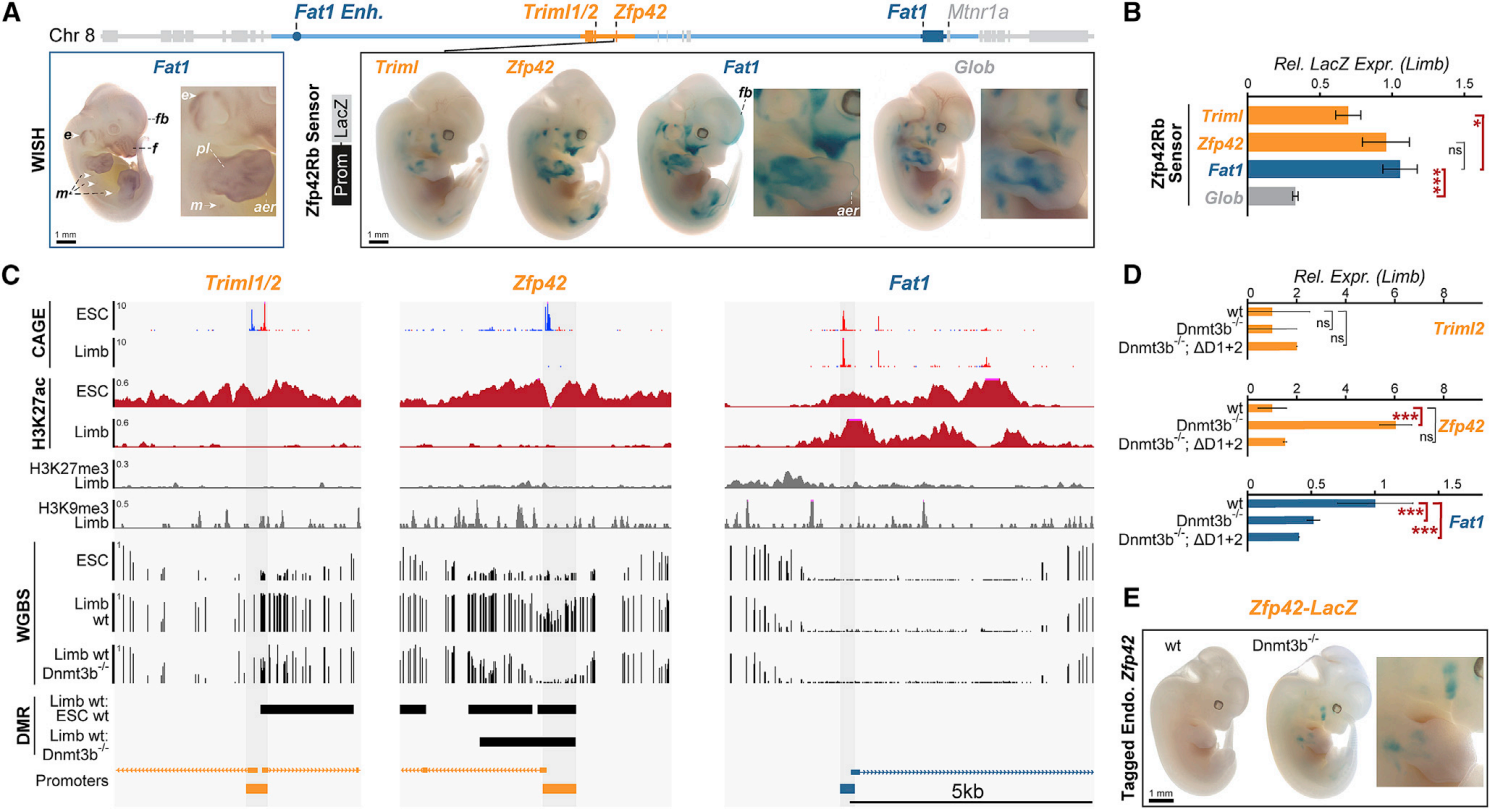
DNA methylation and not enhancer compatibility renders *Zfp42* insensitive to *Fat1* regulatory information (A) E12.5 embryos stained for *Fat1* WISH (left) or LacZ expression (right) driven at Zfp42Rb by the *Triml1/2*, *Zfp42*, *Fat1*, or *β-globin* (*Glob*) core promoters. n = 4–10 embryos. Staining indicated in the ear (e), mammary glands (m), face (f), forebrain (fb), proximal limb (pl), and apical ectodermal ridge (AER). Scale bar, 1 mm. WISH is reproduced from Figure 4. (B) qRT-PCR expression analysis of Promotor-LacZ sensor mRNA in E12.5 limbs. Error bars, SD calculated from 3–8 biological replicates. ^∗∗∗^p < 0.001, ^∗^p < 0.05, and non-significant (ns) from Welch's t test comparisons. (C) CAGE, H3K27ac, H3K27me3, H3K9me3, and WGBS tracks from ESCs and/or E11.5 limb buds. Cloned minimal promoters are highlighted in gray. Differentially methylated regions (DMRs) are denoted by black bars. (D) RNA-seq expression effects of Dnmt3b knockout with or without D1+D2 deletion. Error bars: standard deviation calculated from 3–4 biological replicates. ^∗∗∗^p < 0.001, ^∗^p < 0.05, non-significant (ns). (E) Staining of lacZ-tagged endogenous *Zfp42* in wild-type and DNTM3B^−/−^ E12.5 embryos. Scale bar, 1 mm. See Figure S5 and Tables S1, S2, and S4.

As regulatory information is sampled throughout the intact limb TAD, we postulated that strict functional incompatibility of *Zfp42R* promoters with *Fat1* enhancers maintains their later embryonic inactivity (van Arensbergen et al., 2014). We therefore exchanged the *Zfp42*, *Triml1/2*, or *Fat1* core promoters into the LacZ regulatory sensor and positioned these constructs at *Zfp42*Rb, 20 kb from the endogenous *Zfp42* promoter (Figure 5A). As a control, these modified sensor constructs were first integrated at the *Rosa26* safe harbor locus to confirm their lack of autonomous, enhancer-independent transcription (Figure S5C). In all cases, no LacZ signal was observed at the enhancer-free *Rosa26* locus (Figure S5D). By contrast, the transplanted *Triml1/2*, *Zfp42*, and *Fat1* promoters integrated at *Zfp42*Rb all recapitulated the *Fat1-*like limb, face, and ear LacZ activity pattern observed with the previous *β-globin* sensor (Figure 5A). Thus, remarkably, *Zfp42R* and *Fat1* promoter sequences are compatible with active *Fat1* enhancers in the TAD in later embryos. Nevertheless, differences in *Fat1* enhancers responsiveness were observed. qPCR in embryonic limbs demonstrated the *Glob* and *Triml1/2* promoters drive 66% and 29% less *lacZ* RNA transcription, respectively, than their *Fat1* and *Zfp42* counterparts (Figure 5B). Likewise, the *Fat1* promoter generated additional *Fat1-*expression domains, including the forebrain (fb) and limb apical ectodermal ridge (AER), thereby indicating some degree of selectivity exists (Figure 5A). However, despite this, it is clear that these differences in enhancer-promoter compatibility cannot explain complete *Zfp42R* gene inactivity in later embryos. Instead, this inactivity must be maintained by highly context-dependent promoter silencing.

### DNA methylation desensitizes *Zfp42* to limb enhancers

We thus sought to determine which repressive mechanisms could drive the context-dependent silencing of more recent *Zfp42R* genes in the embryonic limb. Analysis of published ChIP-seq identified no enrichment of H3K27me3 or H3K9me3 at *Zfp42R* promoters in E11.5 limbs, thereby ruling out both polycomb and classical heterochromatization as silencing mechanisms (Figure 5C; Gorkin et al., 2020). However, similar to past reports, our whole genome bisulfite sequencing (WGBS) identified differentially methylated regions (DMRs) between limb buds and ESCs that surround the *Zfp42* and *Triml1*/*2* promoters (Figure 5C; Borgel et al., 2010; Kim et al., 2014). Specifically, the DMRs at the *Zfp42* or *Triml1/2* promoters go from 13%–25% DNA methylation in ESCs to 57%–93% methylation in limb buds. Conversely, matching its on-going transcription, the *Fat1* promoter remains permanently unmethylated in both tissues. Consequently, we reasoned that highly context-specific DNA methylation renders *Zfp42R* genes permanently insensitive to ancient *Fat1*-enhancer activities in later embryonic tissues. Supporting this, bisulfite conversion cloning demonstrated that DNA methylation is lost at the transplanted *Zfp42* promoter when inserted only 20 kb from its endogenous location at Zfp42Rb (Figures S5F and S5G).

We thus generated E11.5 embryos lacking the *de novo* DNA methyltransferase 3B (Dnmt3b) (Figure S5E). WGBS in Dnmt3b^−/−^ embryonic limbs confirmed a DMR denoting a 71% loss of methylation at the *Zfp42* but not *Triml1/2* or *Fat1* promoters, as reported previously (Figure 5C; Borgel et al., 2010). Unfortunately, further reductions to DNA methylation in limbs was not possible as embryos lacking both DNMT3A and DNMT3B died before E11.5, as observed in past reports (data not shown) (Okano et al., 1999). This redundancy meant *Triml1/2*’s still methylated promoter remained transcriptionally repressed in E11.5 Dnmt3b^−/−^ limbs. Nevertheless, *Zfp42* displayed 6-fold upregulation when partially unmethylated and we confirmed this ectopic activity is driven by *Fat1* enhancers (Figure 5D). Specifically, ectopic *Zfp42* limb expression was abolished when most *Fat1* enhancers were removed in double Dnmt3b^−/−^;ΔD1+D2 embryos (Figure 5D). Moreover, tagging the endogenous *Zfp42* with LacZ demonstrated ectopic expression occurs in a *Fat1*-like pattern and only in E12.5 Dnmt3b^−^/^−^ embryos (Figure 5E). Combined, this demonstrates the endogenous *Zfp42* promoter is rendered insensitive to *Fat1* limb enhancers by highly context-dependent silencing that is driven by at least DNMT3B-driven DNA methylation.

Nevertheless, we note two intriguing observations. First, *Zfp42* was only activated in Dnmt3b^−/−^ limbs to 1/25^th^ of *Fat1’s* limb expression and to 1/150^th^ of its maximal potential activity in ESCs. As such, additional redundant silencing, for example, by DNMT3A or alterative repressive mechanisms, likely operate simultaneously. Second, ectopic *Zfp42* activation was associated with a ∼50% reduction in *Fat1* expression, suggesting the competing use of *Fat1* enhancers impairs the latter’s regulation (Figure 5D). However, this cannot be explained by altered enhancer-promoter contacts or TAD structure as neither was observably affected in Dnmt3b^−/−^ limb cHi-C (Figures S5H and S5I).

### Conflicting gene expression is common within multi-gene TADs

**Figure 6:**
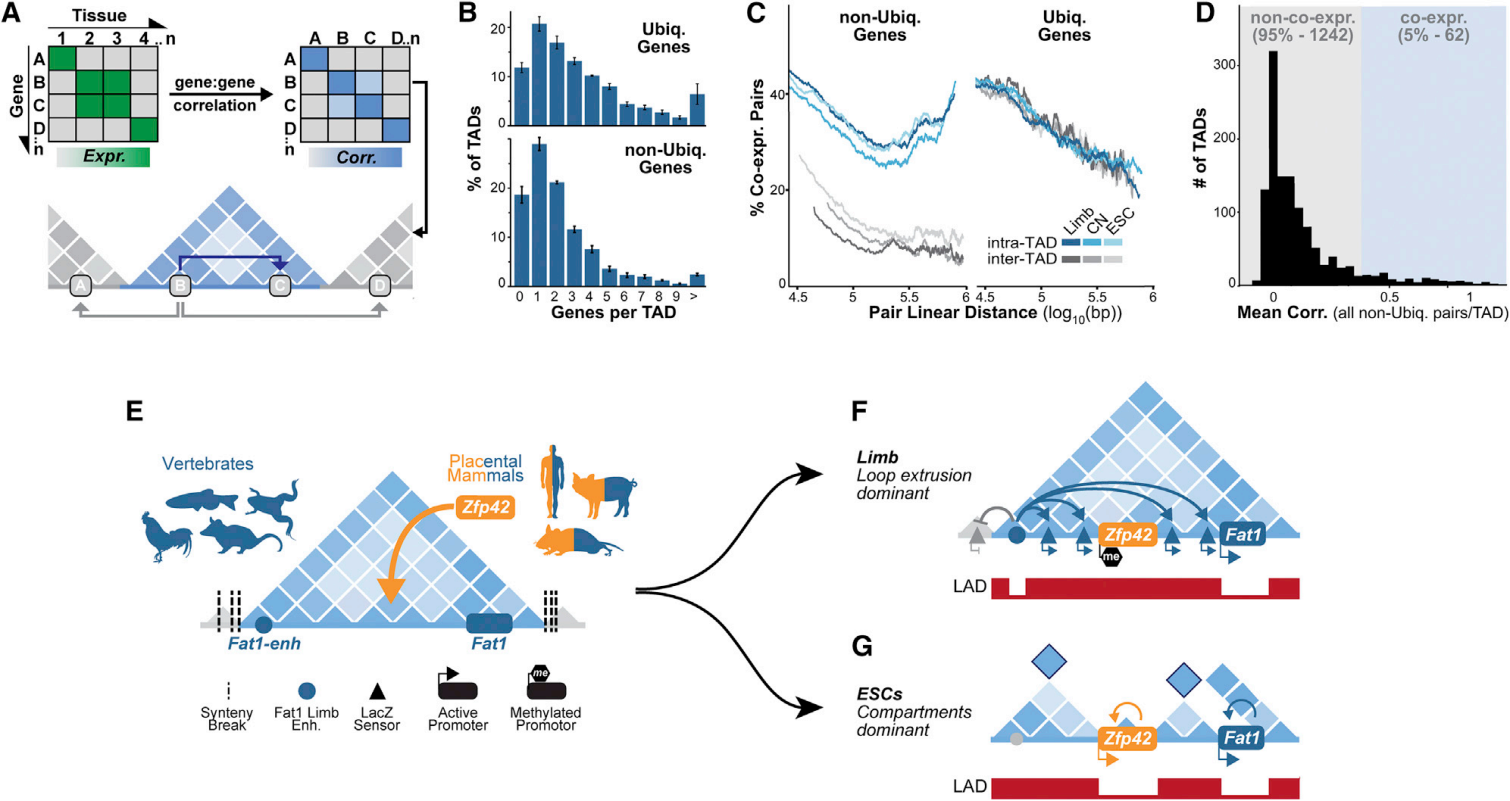
Divergent promoter regulation is common in TADs throughout the genome (A) Summary of TAD co-expression analysis. Gene pair co-expression was determined from FANTOM5 CAGE data, whereas TADs were identified in limb, cortical neuron (CN), and ESC Hi-C (Bonev et al., 2017; Consortium et al., 2014; Kraft et al., 2019; Lizio et al., 2015). (B) Average frequency distribution of non-Ubiq. and Ubiq. genes in TADs. (C) Fraction of co-expressing intra-TAD and inter-TAD gene pairs according to their linear separation. Lines represent a moving window average of 2,000 gene pairs. (D) Frequency distribution of mean expression correlation between all non-Ubiq. genes in a domain for all multi-gene TADs. (E–G) Model for evolution of independent *Zfp42R* and *Fat1* regulation. (E) *Fat1*, its enhancers, and TAD existed together as a regulatory unit in all vertebrates despite frequent flanking synteny breaks. *Zfp42* and *Triml1/2* emerged with independent regulation in placental mammals. (F) In limbs, *Fat1* enhancers emerge from LADs and promiscuously sample promoters throughout the domain's both active and NE-attached inactive compartments. However, despite this and its functional compatibility with *Fat1* enhancers, DNA methylation of *Zfp42’*s promoter prevents its activation. (G) In ESCs, activity-driven compartmentalization and perhaps weakened loop extrusion restructures the TAD, thereby driving the *Zfp42R* and *Fat1* genes to independently utilize only local enhancers. See Figure S6.

**Figure S6:**
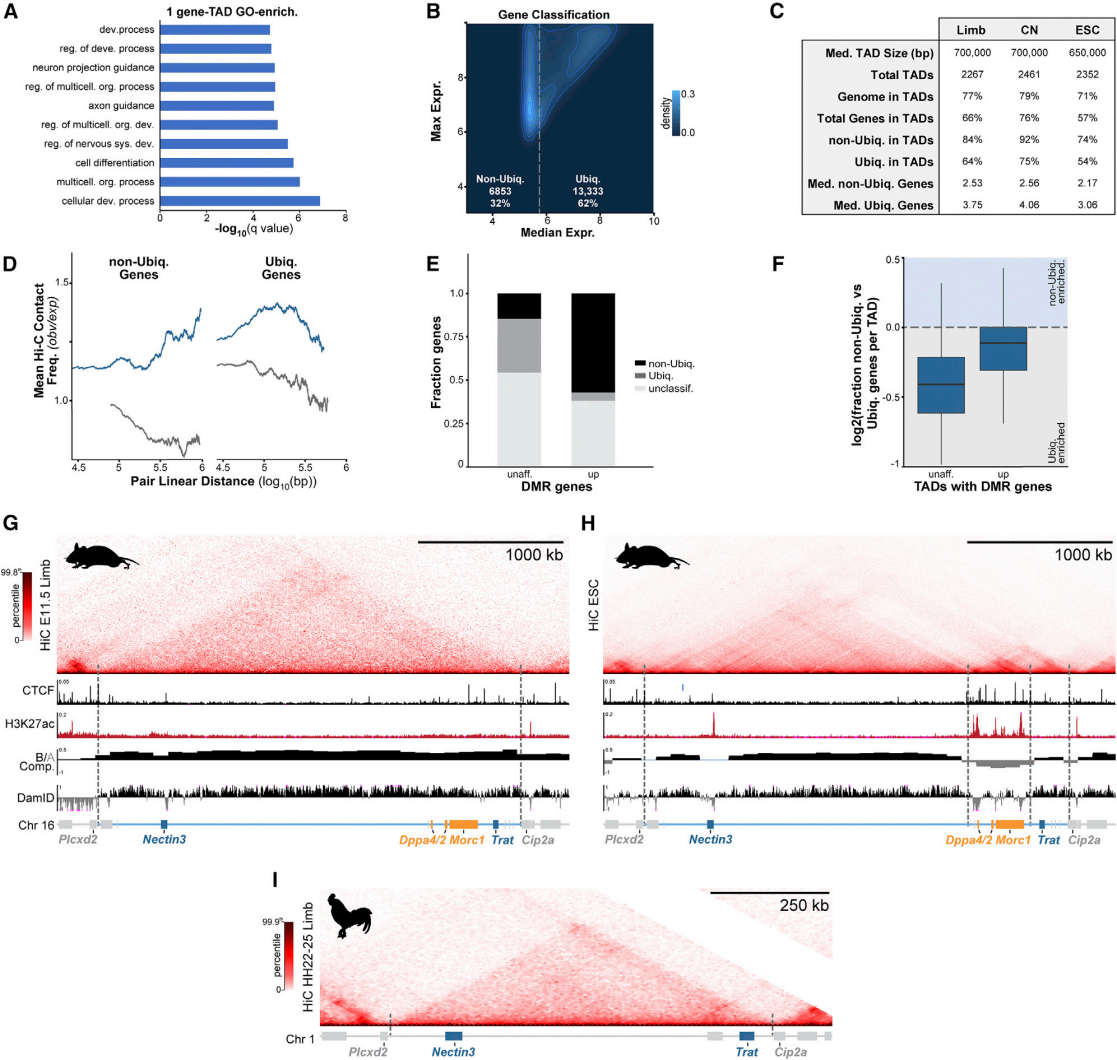
Summaries of co-expression analysis and genome-wide effects of DNA hypomethylation, related to Figure 6 (A) GO-term enrichment for genes within single-gene TADs (Eden et al., 2009). (B) Classification of genes into non-ubiquitously (non-Ubiq.) and ubiquitously (Ubiq.) expressed classes according to their maximum and median expression across FANTOM5 CAGE samples. (C) TAD and gene statistics in limb, CNs and ESCs. (D) Mean observed/expected KR-normalized Hi-C contact frequency between intra-TAD or inter-TAD gene pairs. Lines represent a moving window average of 2,000 gene pairs. Non-Ubiq. gene co-expression strongly correlates with their increased contact frequency within TADs and, in particular, near TAD boundaries. (E) non-Ubiq. and Ubiq. expression classification of genes that possess hypomethylated DMR promoters in DNMT3B^−/−^ limbs. Unclassified reflects genes that were detected in limb RNA-seq but did not pass thresholds for classification into Ubiq. or non-Ubiq. FANTOM5 classes. (F) Fraction of non-Ubiq. versus Ubiq. genes in each TAD of hypomethylated DMR promoters. (G–I) Hi-C at the *Dppa2/4* locus from E11.5mouse limb buds (G), mouse ESCs (H) morphologically stage-matched chicken limb buds (I). Matching CTCF and H3K27ac ChIP-seq, compartments and Lamin B1 DamID tracks are shown below. Dotted lines demarcate partitioned domains. *Nectin3* and *Trat* (dark blue) occupy a large gene desert and TAD (light blue) into which *Morc1* (orange) emerged in tetrapods. *Dppa2* and *4* (orange) emerged later in eutherians. Like *Zfp42R* genes, *Dppa2/4* and *Morc1* are active in ESCs where they are isolated with local enhancers in a separate domain within a disassembled TAD (Sima et al., 2019).

Our results indicate that at least two mechanisms can adapt single regulatory landscapes to host multiple expression programs in evolution. We thus globally quantified how pervasive such conflicting expression is in regulatory landscapes genome wide with available Hi-C and FANTOM5 expression data (Figure 6; Bonev et al., 2017; Consortium et al., 2014; Kraft et al., 2019). We find ∼12% of the ∼2,400 TADs found in several mouse cell types contained only a single gene that were collectively enriched in developmental GO-terms (Figure S6A; Eden et al., 2009). Thus, as previously suggested, a fraction of developmental loci are isolated alone within dedicated mono-gene TAD regulatory landscapes (Wu et al., 2021). Nevertheless, ∼88% of TADs contained multiple genes which we classified into ubiquitous (Ubiq.) or non-ubiquitous (non-Ubiq.) expression classes (Figures 6B, S6B, and S6C) (see STAR Methods). Thus, multi-gene TADs like the *Zfp42/Fat1* domain dominate in the genome and frequently contain multiple non-Ubiq. “developmental” and/or Ubiq. “housekeeping” genes.

We thus determined if multi-gene TADs support co-ordinated or divergent gene activities through co-expression analysis. We find so-called “developmental” non-Ubiq. genes, but not their “housekeeping” Ubiq. counterparts, were more frequently co-expressed when located within the same TAD, similar to past reports (Figure 6C; Flavahan et al., 2016; Nora et al., 2012; Shen et al., 2012; Zhan et al., 2017). Significantly, such gene co-expression correlated with the higher Hi-C contact frequency that occurs within TAD boundaries, suggesting that it is a product of increased shared enhancer interactions (Figure S6D). Despite this, most non-Ubiq. genes sharing a TAD are not co-regulated, and hence, only 5% of TADs display high mean co-regulation between all their hosted non-Ubiq. genes (Figure 6D) (see STAR Methods). Thus, like *Zfp42*/*Fat1*, conflicting developmental gene expression within shared TAD regulatory landscapes is a pervasive feature of the genome during evolution.

We finally tested the extent to which DNA methylation resolves these pervasive regulatory conflicts by expanding our analysis of Dnmt3b^−/−^ limbs. We detected 594 promoters displaying hypomethylated DMRs that displayed distinct transcriptional responses to hypomethylation (42 upregulated, 4 downregulated, 546 unaffected). As we predicted, these behaviors were linked to a DMR promoter’s expression class and TAD environment. Upregulated DMR promoters were enriched in non-Ubiq. “developmental” genes and were located in TADs that contain a higher fraction of other non-Ubiq. genes (Figures S6E and S6F). By contrast, unaffected DMR promoters were enriched in Ubiq. “housekeeping” genes and were located within TADs that contain fewer non-Ubiq. genes. Thus, like *Zfp42*, lost DNA methylation preferentially activates promoters if they are (1) developmental and (2) exposed to the enhancers of other developmental loci in the same TAD.

Together, this indicates that regulatory conflicts arise frequently as genes emerge or are rearranged into shared domains during evolution. However, such conflicts can be resolved by DNA methylation-driven silencing, 3D restructuring, and likely additional cooperating mechanisms (Figures 6E–6G).

## Discussion

TADs are frequently described as stable and conserved structural scaffolds that ensure transmission of enhancer activities to promoters found within a domain’s boundaries (Andrey and Mundlos, 2017). In this simple model, genes with similar functions can be controlled together in a shared TAD, whereas those requiring divergent regulation must be placed alone in separated domains (Wu et al., 2021). However, we find most TADs in the genome contain multiple independently regulated developmental genes as seen in previous studies of specific loci (e.g., the essential *Hox* genes) (Andrey et al., 2013; Huang et al., 2017; Noordermeer et al., 2011; Palstra et al., 2003; Soshnikova and Duboule, 2009). Consequently, a simple TAD regulation model cannot alone explain genome evolution. Instead, we show multiple mechanisms incorporated *Zfp42R* gene regulation without disrupting the pre-existing *Fat1* landscape and its diverse reported physiological functions (Figures 6E–6G; Peng et al., 2021). As such, even single loci can easily incorporate conflicting regulatory programs in evolution and this capacity equips the genome with enormous regulatory complexity and flexibility. In human genetics, this also likely explains why many genomic rearrangements that create new enhancer-promoter combinations in shuffled TADs do not drive gene misexpression or disease (Despang et al., 2019; Laugsch et al., 2019). Specifically, because other mechanisms—including 3D-restructuring and context-dependent silencing—refine enhancer usage in regulatory landscapes.

We reveal that even evolutionarily stable TADs can be massively restructured to regulate transcription in specific cell types. However, unlike previous examples, we show this is unexpectedly driven independently of cohesin and CTCF by underlying chromatin activity (Bonev et al., 2017; Isoda et al., 2017). Specifically, the activity of the *Zfp42* and *Fat1* regions in ESCs drives the antagonistic tendency of active and inactive chromatin to spatially separate. As such, both genes become separated with independent enhancers in isolated domains. Intriguingly, other loci reportedly show similar activity-dependent isolation within larger existing TADs, including *Dppa2/4*, that we find also emerged in a pre-existing domain in placental mammals (Figures S6G–S6I; Sima et al., 2019). Thus, TADs are structured by compartmentalization as well as loop extrusion, and this can be altered for evolutionary adaptation. However, this further raises the exciting generalizable possibility that chromatin structure and underlying epigenetic state could both reciprocally drive and self-reinforce one another to control transcription.

We demonstrate that chromatin positioning at the NE in LADs need not have a deterministic role in regulating endogenous gene expression. Several groups have previously ectopically transplanted promoters to or from LADs *in vitro* and revealed the NE is a generally transcriptionally repressive environment (Finlan et al., 2008; Leemans et al., 2019; Reddy et al., 2008). Accordingly, retaining specific genes at the NE reportedly maintains their inactivity during *in vitro* differentiation (Poleshko et al., 2017; Robson et al., 2016). However, we find that *Fat1* enhancers can still activate LacZ reporter genes despite their integration in LADs and extensive intervening lamina-association separating them. Thus, LADs are neither (1) sufficient to silence genes nor (2) effective insulators of enhancer-promoter communication. Instead, LADs can likely be readily restructured to allow genes to locally escape and activate when needed (Brueckner et al., 2020; Therizols et al., 2014). As such, LADs are not entirely inhospitable environments for gene regulation or the emergence of novel gene activities in evolution.

Instead, we find that extreme differences in expression in multi-gene TADs can be driven by a promoter’s exact endogenous position and sequence-context rather than its incompatibility with specific enhancers. Specifically, the *Triml1/2 and Zfp42* promoters can be activated by embryonic *Fat1* enhancers in their TAD, *but* only when relocated away from their endogenous positions. This is significant as the extent to which enhancer-promoter compatibility regulates mammalian transcription remains controversial and is largely examined outside of native contexts in episomal *in vitro* assays (Bergman et al., 2021; Martinez-Ara et al., 2022; Ray-Jones and Spivakov, 2021; van Arensbergen et al., 2014). As result, such approaches may fail to predict many endogenous gene expression outcomes found in native regulatory landscapes (Arnold et al., 2017; Martinez-Ara et al., 2022).

Last, we find that DNA methylation can drive context-dependent silencing but is not only targeted to genes by promoter sequence. Instead, we find promoter DNA methylation can be dependent on genomic position and, presumably, the sequence of the adjacent flanking DNA. We thus extend the features that target DNA methylation beyond the absence/presence of specific chromatin modifications (Ooi et al., 2007; Weinberg et al., 2021; Zhang et al., 2010). However, in doing so, we also demonstrate an additional means through which DNA methylation controls genes, not by suppressing a promoter’s intrinsic transcriptional activity but instead its sensitivity to enhancers. Importantly, this may explain why only minor gene expression defects are observed when DNA methylation is eliminated entirely in early embryos (Grosswendt et al., 2020; Yagi et al., 2020). In this view, misexpression would be limited to only unmethylated genes exposed to enhancers within shared landscapes and, even then, only in the specific cell types where those enhancers are active. However, we note that our inability to entirely eliminate DNA methylation and survey transcription in all cell types prevents testing the full extent of this regulation. Nevertheless, DNA methylation adds to the repressive mechanisms known to refine promiscuous enhancer usage within multi-gene TADs (Gjaltema et al., 2021; Noordermeer et al., 2011; Soshnikova and Duboule, 2009).

### Limitations of the study

We find that regulatory conflicts are a generalizable feature of evolving genomes and are resolved by surprisingly diverse mechanisms. However, it will be necessary to determine the extent to which these or other mechanisms resolve the extensive regulatory conflicts that we observe at a genome-wide scale. Combining recent enhancer-promoter models with cell-type-specific measurements of promoter state and 3D structure will greatly aid this (Fulco et al., 2019; Nasser et al., 2021; Zuin et al., 2021). Moreover, doing so will be particularly significant for human genetics where we cannot reliably predict which patient genomic rearrangements will be benign or will create pathogenic regulatory conflicts. A second essential next step will be to also identify which exact DNA sequences and factors direct context-dependent promoter DNA methylation. Third, we did not observe new enhancer-promoter contacts when *Zfp42* was demethylated and ectopically activated. It will thus be important to elucidate if this is due to the technical resolution limits of cHi-C or because enhancers require only weak and transient promoter contact to activate genes. Last, although not accounting for divergent *Fat1-Zfp42* activity, we find promoters that can display at least 3-fold differences in sensitivity to enhancers in the same TAD. It will be critical to determine the molecular basis of these different promoter sensitivities, and most importantly, if they meaningfully resolve regulatory conflicts in other multi-gene TADs genome wide (Long et al., 2016; van Arensbergen et al., 2014).

## STAR★Methods

### Key resources table

**Table:** 

REAGENT or RESOURCE	SOURCE	IDENTIFIER
**Antibodies**		

rabbit anti-H3K27ac	Diagenode	C15410174
rabbit anti-H3K4me1	Diagenode	C15410037
rabbit anti-H3K27me3	Merck Millipore	07-449
rabbit anti-H3K4me3	Merck Millipore	07-473
rabbit anti-lamin B1	Abcam	ab160486
donkey anti-rabbit IgG-Atto647	Sigma Aldrich	40839
Anti-DIG-AP, 150 U	Roche Diagnostics	11093274910
rabbit anti-DNMT3A	Abcam	ab188470 lot GR224165-2
rabbit antiDNMT3B	Cell Signaling	cs48488, lot 1
rabbit antiACTIN	Sigma Aldrich	A2066

**Bacterial and virus strains**		

One Shot TOP 10 Chemically Competent Cells E.c.	Thermo Fisher	C404006

**Chemicals, peptides, and recombinant proteins**		

Advantage cDNA polymerase	Clontech	639105
Agencourt AMPure XP magnetic beads	Beckman Coulter	A63880
Auxin	Abcam	ab14642
Biozym Blue S'Green qPCR Kit Separate ROX	Biozym	331416S
BCIP, 3ml (150 mg)	Roche Diagnostics	11383221001
Biotin-14-dATP-50 nmol	Thermo Fisher Scientific	19524016
BM-Purple, AP-Substrat	Roche Diagnostics	11442074001
cOmplete, Mini, EDTA-free Protease Inhibitor Coctail	Sigma Aldrich	4693159001
Covaris micro TUBE AFA Fiber Pre-Slit Snap-Cap tubes	Covaris	SKU - 520045
DMEM, high glucose, no glutamine	Thermo Fisher	11960085
DNA Pol. Large Fragm. (Klenow)	New England Biolabs	M0210L
Dnase,recombinant,RNase-free (10000 U)	Roche Diagnostics	4716728001
DpnI, recombinant	New England Biolabs	R0176L
DpnII, recombinant	New England Biolabs	R0543S
Dynabeads MyOne Streptavidin T1-10 mL	Thermo Fisher Scientific	65602
ESGRO(LIF)	Millipore	ESG1107
Formamide deionized for molecular biology	PanReac AppliChem	APP A2156,1000
FuGENE HD Transfection Reagent	Promega	E2311
Gelatin 2% solution from bovine skin cell	Sigma Aldrich	G1393
Heparin sodium salt	Sigma Aldrich	H3149
Hygromycin B (50mg/ml)	Thermo Fisher	10687010
Knockout DMEM-500 ml	Thermo Fisher	10829018
L-glutamine (200mM)	Lonza	882027-12
Lent-X concentrator	Takara	631232
Library Efficiency DH5a Competent Cells	Thermo Fisher	18263012
Lipofectamine 2000 Transfection Reagent	Thermo Fisher Scientific	11668019
MEM Non Essential Amino Acids Solution	Thermo Fisher	11140068
NBT, 3ml (300 mg)	Roche Diagnostics	11383213001
NEBNext High-Fidelity 2X PCR Master Mix	New England Biolabs	M0541S
NEBNext Multiplex Oligos for Illumina	New England Biolabs	E7335, E7500
NEBNext Quick Ligation Reaction Buffer (5X)	New England Biolabs	B6058S
NEBNext Ultra II Q5 Master Mix	New England Biolabs	M0544L
Opti-MEM I Reduced Serum Medium, GlutaMAX Supplement	Thermo Fisher	51985026
Penicillin/Streptomycin	Fisher Bioreagents	10003927
Proteinase K	Roche Diagnostics	1000144
Puromycin	SIGMA-ALDRICH	P8833
Recombinant Human/Mouse FGF-8b Isoform	R&D Systems	#423-F8-025/CF
Recombinant Mouse Wnt-3a protein	R&D Systems	#1324-WN-010/CF
Ribonuclease A from bovine pancreas, Type 1-A, RNase A	Sigma Aldrich	R4875
Rnase Inhibitor (2000 U)	Roche Diagnostics	3335399001
Roti-Phenol/ Chloroform/ Isoamylalcohol	Carl Roth	A156.2
SP6-RNA Polymerase (1000 U)	Roche Diagnostics	10810274001
SYBR Green I	Thermo Fisher	S7563
T4 DNA Ligase	New England Biolabs	M0202L
T4 DNA Polymerase	New England Biolabs	M0203L
T4 Polynucleotide Kinase NK	New England BioLabs	M0201
T7-RNA Polymerase (1000 U)	Roche Diagnostics	10881767001
Tagment DNA Buffer	Illumnia	15027866
Tagment DNA Enzyme 1 (TDE1)	Illumnia	15027865
tRNA from Baker's Yeast	Sigma Aldrich	R6750
Trypsin-EDTA (0.05%), phenol red	Thermo Fisher	25300096
Water for Injection (WFI) for cell culture	Thermo Fisher	A1287303
X-beta-Gal min 99 %, BioScience-Grade	Carl Roth	2315.3
Cot-1 DNA	Invitrogen Life Technologies	18440-016

**Critical commercial assays**		

0.45 μm2 low protein-binding PES syringe filter	Millipore	SLHP003RS
Accel-NGS Methyl-seq DNA library kit	Zymo	DL-ILMMS-12
Agencourt AMPure XP beads	Beckman Coulter	A63881
EpiTect Bisulfite Kit	QIAGEN	59104
Dig-RNA-labeling Mix	Roche Diagnostics	11277073910
DNA Clean & Concentrator-5 kit	Zymo	D4013
Dneasy Blood & Tissue Kit(50)	QIAGEN	69504
EpiTect Bisulfite Kits	QIAGEN	N/A
EZ DNA Methylation-Gold Kit	Zymo	D5005
iDeal ChIP-seq kit for histones	Diagenode	C01010051
KAPA HyperPrep kit for NGS DNA Library Prep	Roche	7962363001
MinElute PCR Purification Kit	QIAGEN	28004
MinElute Reaction Clean up kit	QIAGEN	28206
MycoAlert Assay Control Set	Lonza	LT07-518
MycoAlert detection kit	Lonza	LT07-118
NEBNext Multiplex Oligos for Illumina kit	New England Biolabs	E7500
PureLink Genomic DNA Mini Kit	Thermo Fisher	K182002
Quick Ligation™ Kit	New England Biolabs	M2200S
Rneasy Mini Kit	QIAGEN	74104
Vectashield	Vector laboratories	H-1000
Zymo DNA Clean & Concentrator-5 kit	Zymo	D4013
Zymo Quick-DNA/RNA Microprep Plus Kit	Zymo	D7005
FISH Tag DNA Kit	Invitrogen Life Technologies	F32951

**Deposited data**		

Raw and processed sequencing data	This study	GEO: GSE185775
Whole genome bisulphite sequencing	This study	GEO: GSE185765
RNA-Seq in wildtype & mutant mouse E11.5 limbs and ESCs	This study	GEO: GSE185766
Hi-C in chicken and mouse embryonic limbs	This study	GEO: GSE185768
DamID-seq in wildtype mouse E11.5 limbs and ESCs	This study	GEO: GSE185771
ChIP-seq in wildtype mouse ESCs	This study	GEO: GSE185772
ATAC-seq in wildtype mouse E11.5 limbs	This study	GEO: GSE185774
HiC in mouse ESCs after transcription inhibition	Hsieh et al., 2020	4DNES14CNC1I
Hi-C in mouse ESCs and Cortical Neurons	Bonev et al., 2017	GEO: GSE96107
Hi-C in mouse E11.5 limb buds	Kraft et al., 2019	GEO: GSE116794
ChIP-seq for CTCF, Rad21 and H3K9me3 in mouse ESCs and E11.5 limb buds	Kraft et al., 2019	GEO: GSE116794
ChIP-seq for H3K4me1, H3K4me3, H3K27ac and H3K27me3 in E11.5 limb buds	Andrey et al., 2017	GEO: GSE84795
ATAC-seq in mouse ESCs	Bauer et al., 2021	GEO: GSE157448
Fantom5 CAGE Expression datasets	Lizio et al., 2015	https://fantom.gsc.riken.jp/5/data/
DamID in mouse E11.5 limb cells	Allou et al., 2021	GEO: GSE137335
Hi-C in mouse inner cell mass and 8-cell embros	Du et al., 2017	GEO: GSE82185
Hi-C and H3K27ac & CTCF ChIP-seq in human ESCs and cardiomyocytes	Zhang et al., 2019	GEO: GSE116862
Hi-C in 48 hr hpf Zebrafish	Yang et al., 2020	GEO: GSE134055
Hi-C in xenopus brain	Niu et al., 2021	SRA: PRJNA606649
Hi-C in pig embryonic fibroblasts	Li et al., 2020	GEO: GSE153452
DamID in human RPE and ESCs	van Schaik et al., 2020	4D nucleome
ChIP-seq for H3K36me3 in mouse ESCs	Encode	GEO: GSE31039
ChIP-seq for CTCF in chicken	Jerković et al., 2017	GEO: GSE86089
scRNA-seq in gastrulating E6.5-8.5 mouse embryos	Pijuan-Sala et al., 2019	ArrayExpress: E-MTAB-6967
scRNA-seq in E9.5-E12.5 mouse embryos	Cao et al., 2019	GEO: GSE119945
scRNA-seq in E9.5-E14.5 mouse placentas	Marsh and Blelloch, 2020	GEO: GSE152248

**Experimental models: Cell lines**		

G4 ESCs (XY, 129/Sv x C57BL/6 F1 hybrid)	Georg et al., 2007	N/A
CTCF-AID-GFP E14 ESCs	Nora et al., 2017	N/A
Rad21-AID-GFP E14 ESCs	Liu et al., 2021	N/A
^∗^mutant ESC lines are listed in Table 1	This study	N/A
293FT	Thermofisher	R70007

**Experimental models: Organisms/strains**		

Wild-type and mutant mice derived from G4 ESCs	This study	N/A
Opossums *(Monodelphis domestica)*	Naturkunde Museum, Berlin	N/A
Chicken (*Gallus Gallus*)	Valo Biomedia	N/A

**Oligonucleotides**		

Zfp42/Fat1 cHi-C libary	This study	mm10, chr8: 39022300-48000000
DamID oligos and primers see Table S2	Vogel et al., 2007	N/A
WISH probe primers see Table S2	This study	N/A
Genotyping primers see Table S2	This study	N/A
Cloning primers see Table S2	This study	N/A
Oligopaint probes see Table S3	This study	N/A

**Recombinant DNA**		

pLGW-Dam-V5-Lamin B1 (Mm)	Steensel Lab	N/A
pLGW-V5-Dam	Steensel Lab	N/A
pMD2.G	Bird Lab	N/A
psPAX2	Bird Lab	N/A
BAC for *Fat1R*	CHORI/BACPAC	RP23-451E23
pX459 pSpCas9(BB)-2A-Puro vector	Addgene	#62988
Fat1 promoter 302bp	This study	chr8: 44935221 - 44935522
Zfp42 promoter 602bp	This study	chr8: 43306912 - 43307513
Triml promoter 427bp	This study	chr8: 43180161 - 43180587
Fat1 enhancer	This study	chr8: 41591354 - 41594915
Knockin donor vectors & corresponding pX459 sgRNAs see Table S1	This study	N/A

**Software and algorithms**		

CRISPR design	https://www.benchling.com	N/A
R	https://www.r-project.org	N/A
MACS2.0	https://github.com/taoliu/MACS	N/A
Bowtie2	Langmead and Salzberg, 2012	N/A
Samtools	http://samtools.sourceforge.net	N/A
HiCUP v0.8.1	Wingett et al., 2015	N/A
Cooltools	https://zenodo.org/record/5214125	N/A
Juicer	Durand et al., 2016	N/A
Genrich	https://github.com/jsh58/Genrich/	N/A
UCSC genome browser	https://genome.ucsc.edu	N/A
WashU browser	https://epigenomegateway.wustl.edu	N/A
HMMt	https://github.com/gui11aume/HMMt	N/A

**Other**		

FISH and SBS-modelling statistics summary see Table S5	This study	N/A
List of bridging species for conservation analysis see Table S6	This study	N/A

### Resource availability

#### Lead contact

Further information and requests for resources and reagents should be directed to, and will be fulfilled by, the lead contact Michael I. Robson (robson@molgen.mpg.de).

#### Material Availability

All unique/stable reagents generated in this study are available from the lead contact without restriction.

### Experimental model and subject details

Mouse G4 ESCs (XY, 129S6/SvEvTac x C57BL/6Ncr F1 hybrid) were grown as described previously on a mitomycin-inactivated CD1 mouse embryonic fibroblast feeder monolayer on gelatinised dishes at 37^o^C, 7.5% CO_2_ (Andrey and Spielmann, 2017; George et al., 2007). CTCF-AID-GFP and Rad21-AID-GFP E14 ESCs were cultured feeder-free on gelatinised dishes at 37^o^C, 7.5% CO_2_. All ESCs were cultured in ESC medium containing knockout DMEM with 4,5 mg/ml glucose and sodium pyruvate supplemented with 15% FCS, 10 mM Glutamine, 1x penicillin/streptomycin, 1x non-essential amino acids, 1x nucleosides, 0.1 mM beta-Mercaptoethanol and 1000 U/ml LIF. Medium was changed every day while G4-cells were split every 2-3 days or were frozen at 1x 10^6^ cells/cryovial in ESC medium containing 20% FCS and 10% DMSO. ESCs and feeder cells were tested for Mycoplasma contamination using the MycoAlert detection kit and MycoAlert Assay Control Set.

E11.5 limb cells were isolated from C57BL/6 embryonic limbs through trypsinization, filtration (40 μm) and centrifugation. Cell suspensions were then plated on gelatine-coated plates at 37^o^C in 5.0% CO2 in DMEM/F12 supplemented with 10% FCS, 4 mM L-Glutamine, 1x penicillin/streptomycin, 250 ng/ml Recombinant Mouse Wnt-3a protein and 150 ng/ml Recombinant Human/Mouse FGF-8b Isoform.

Mutant embryos and mutant live animals were produced through tetraploid or diploid aggregation, respectively (Artus and Hadjantonakis, 2011). Female mice of the CD1 strain were used as foster mothers. Mutant lines were established and maintained by crossing with wildtype C57Bl.6/J animals. All mice were housed in a centrally controlled environment with a 12 h light and 12 h dark cycle, temperature of 20–22.2 °C, and humidity of 30–50%. Bedding, food and water were routinely changed. All animal procedures were conducted as approved by the local authorities (LAGeSo Berlin) under the license numbers G0176/19, G0247/13 and G0243/18.

HH22 and HH24 Chicken embryos were extracted from fertilised chicken eggs (Valo Biomedia) incubated at 37.8^o^C, 45% humidity.

Embryonic stages of opossum originated from the breeding colony of *Monodelphis domestica* maintained under permit ZH104 (issued by the local authority, LAGeSo) in the animal care facility of the Museum für Naturkunde, Berlin. All opossums were housed in a centrally controlled environment with a reversed 12 h dark and 12 h light cycle, temperature of 24–26 °C, and humidity of 60-65%. Bedding, food and water were routinely changed. Females were euthanized using an overdose of Isoflurane under T0198/13 (issued by LAGeSo) according to national and international standards. Samples were taken immediately after death was confirmed. To culture opossum embryonic fibroblasts (OEFs), stage 30 embryos were isolated and the heads removed by dissection. Following, cells were isolated through trypsinization, filtration (100 μm) and centrifugation. Cell suspensions were then plated on gelatine-coated plates at 37^o^C in 5.0% CO2 in ESGRO Complete Basal Medium with supplement until a stable line of OEFs was established.

### Method details

#### Plasmid Construction

SgRNAs were designed at desired structural variant breakpoints or knockin sites using the Benchling design tool (https://www.benchling.com/). Complementary sgRNA oligos were subsequently annealed, phosphorylated, and cloned into the BbsI site of dephosphorylated pX459 pSpCas9(BB)-2A-Puro vector (Addgene; #62988). For insertion of lacZ sensors, asymmetric homology arms surrounding insertion sites were first synthesised with a multiple cloning site that bisected, and so inactivated, the sgRNA. Once homology arms were cloned into a vector, the lacZ sensor insert harbouring the *β-globin* minimal promoter and polyA terminator were subsequently inserted by restriction digest (Symmons et al., 2014). For testing alternative promoters, the *β-globin* promoter was substituted for synthesized or PCR-amplified *Zfp42, Triml1/2*, or *Fat1* promoters through restriction cloning. In all cases, core promoters incorporate at least 250 bp upstream and 50 bp downstream of the major endogenous TSS-defined in FANTOM5 CAGE transcriptomes (Figure 5C; key resources table). The bidirectional *Triml1/2* promoter was inserted to enable lacZ transcription from the *Triml2* orientation. For enhancer lacZ reporter experiments, the mouse *Fat1-*enh sequence was PCR-amplified and inserted into a phosphoglycerate kinase (PGK) promoter targeting vector containing FRT sites for insertion into C2 ESCs. A list of sgRNAs, corresponding homology constructs and resulting mutant ESCs can be found in Table S1. Cloned enhancer and promoter sequences can be found in the key resources table. All plasmids are available on Addgene.

#### CRISPR-mediated genome editing

CRISPR was subsequently performed as described previously (Kraft et al., 2015). Briefly, 300,000 G4 ESCs (George et al., 2007) were seeded on CD1 feeders 16 h prior to transfection. For structural variants, ESCs were transfected with 4 μg of both sgRNAs targeting each breakpoint using FuGENE HD according to manufacturer’s instructions. For site-specific knockins, ESCs were transfected with 8 μg of the sgRNA and 4 μg of the homology construct. After 24 h, transfected cells were transferred onto puromycin-resistant DR4 feeders and treated with puromycin for 48 h. ESCs were grown for a further 4-6 days after which colonies were picked and transferred to CD1 feeders in 96-well plates. Plates were subsequently split into triplicates after 2-3 days, two for freezing and one for DNA harvesting. Following lysis and genotyping, selected clones were expanded from frozen plates after which genotypes were reconfirmed. Potential structural variant and knockin ESC clones were first identified by PCR-detection of unique deletion breakpoints or site-specific insertion breakpoints, respectively. Desired homozygous or heterozygous copy number were then determined by qPCR. All cell lines and corresponding genotyping primers can be found in Tables S1 and S2.

#### Enhancer Reporter Line Generation

The flippase (FLP)-flippase recognition target (FRT) system was used to introduce enhance-LacZ reporter constructs into C2 ESCs. This modified ESC line contains a phosphoglycerate kinase neomycin selection cassette flanked by FRT sites and a promoter- and ATG-less hygromycin cassette targeted downstream of the *Col1A1* locus (Beard et al., 2006). 800,000 C2 ESCs were seeded onto a feeder-coated 6-well plate and transfected with 9 μg of targeting construct, 3 μg FLP-encoding vector, 1 μl Lipofectamine LTX Plus reagent (Thermo Fisher Scientific), 20 μl Lipofectamine LTX in a to a final OptiMEM volume 250 μl. After 24 h, transfected C2 cells were transferred onto hygromycin-resistant DR4 feeders and treated with hygromycin B (final concentration 150 μg/ml) in ES growth medium for 5-10 days. Colonies were then picked and transferred to CD1 feeders in 96-well plates. Plates were subsequently split into triplicates after 2-3 days, two for freezing and one for DNA harvesting. Following lysis and genotyping, selected clones were expanded from frozen plates after which genotypes were reconfirmed. Genetically modified C2 ESCs were used to produce embryos through diploid aggregation, and genotyping confirmed the presence of the desired mutations in the cells and later in the embryos. Enhancer reporter cell lines and corresponding genotyping primers can be found in Tables S1 and S2.

#### Auxin induced CTCF and Rad21 depletion

Available CTCF-AID-GFP and Rad21-AID-GFP E14 ESCs were treated with 500 μM auxin for 48 h and between 1-6 h, respectively (Liu et al., 2021; Nora et al., 2017). Successful depletion was confirmed through lost GFP signal by FACS. For CTCF-AID-GFP ESCs, bulk cell populations were plated on coverslips for FISH or directly fixed for cHi-C. For cHi-C on Rad21-AID-GFP ESCs, auxin-treated G1 cells were isolated by FACS following fixation and lysis for cHi-C and subsequent DAPI staining. For FISH on Rad21-AID-GFP ESCs, depleted cells were plated on coverslips following 2 h auxin-treatment where only modest changes to cell-cycle had occurred.

#### Western blot

2 million mESCs were collected and then washed twice in PBS. The cell pellet was then resuspended in cell lysis buffer (2 5mM HEPES pH7.6, 5 mM MgCl2, 25 mM KCl, 0.05 mM EDTA, 10% Glycerol, 0.1% IGEPAL, 1X Roche protease inhibitor, 1mM DTT). Nuclei were pelleted from the cell lysate by centrifugation for 5 minutes at 1500 rpm. The nuclei were then washed once (10mM HEPES pH7.6, 3 mM MgCl2, 100 mM KCl, 0.01 mM EDTA, 10% glycerol, 1X Roche protease inhibitor, 1 mM DTT) and centrifuged at 3000g for 5 minutes. Nuclei were then resuspended in 150 μl RIPA Buffer and vortexed for 20 minutes at 4°C. This mixture was spun at 12,000 rpm for 15 minutes and the supernatant was collected for blotting. Western blots were performed with anti-Dnmt3a (1:2000) and anti-Dnmt3b (1:1000) and imaged using HRP chemiluminescence.

#### Tetraploid morula complementation

Mutant ESCs were seeded on CD1 feeders, grown for 2 days and then subjected to diploid or tetraploid aggregation, as previously described (Artus and Hadjantonakis, 2011). CD1 female mice were used as foster mothers. Genotypes of resulting embryos or animals was determined by genotyping PCR as performed in originating ESCs.

#### Whole mount *in situ* hybridization

mRNAs were detected in embryos by WISH using digoxigenin-labelled antisense RNA probes transcribed from cloned mouse, opossum and chicken genomic sequences. Whole embryos were fixed overnight in 4% PFA/PBS, washed in PBS-Tween (PBST; 0.1% Tween) and then dehydrated for at least 10 min each in 25%, 50% and 75% methanol/PBST. Embryos were finally stored at −20°C in 100% methanol. For staining, embryos were rehydrated on ice in reversed methanol/PBST steps, washed in PBST, bleached in 6% H2O2/PBST for 1 h on ice. Following washing in PBST, embryos were then treated with 10 μg/ml proteinase K/PBST for 3 min, incubated in glycine/PBST, washed in PBST, and finally refixed for 20 min in 4% PFA/PBS, 0.2% glutaraldehyde, and 0.1% Tween 20. Following washing in PBST, embryos were incubated at 68°C in L1 buffer (50% deionized formamide, 5× saline sodium citrate, 1% SDS, 0.1% Tween 20 in diethyl pyrocarbonate, pH 4.5) for 10 min. Embryos were then incubated for 2 h at 68°C in hybridisation buffer 1 (L1 with 0.1% transfer RNA and 0.05% heparin). Afterwards, embryos were incubated overnight at 68 °C in hybridisation buffer 2 (hybridisation buffer 1 with 0.1% transfer RNA and 0.05% heparin and 1/500 digoxigenin probe). After overnight hybridisation, unbound probe was removed by 3 x 30 minute washing steps at 68°C in L1, L2 (50% deionized formamide, 2 × saline sodium citrate pH 4.5, 0.1% Tween 20 in diethyl pyrocarbonate, pH 4.5), and L3 (2 × saline sodium citrate pH 4.5, 0.1% Tween 20 in diethyl pyrocarbonate, pH 4.5). Subsequently, embryos were treated for 1 h with RNase solution (0.1 M NaCl, 0.01 M Tris pH 7.5, 0.2% Tween 20, 100 μg/ml RNase A in H2O), followed by washing in Tris-buffered saline, 0.1% Tween 20 (TBST 1) (140 mM NaCl, 2.7 mM KCl, 25 mM Tris-HCl, 1% Tween 20, pH 7.5). Embryos were then blocked for 2 h at room temperature in blocking solution (TBST 1 with 2% fetal bovine serum and 0.2% bovine serum albumin (BSA)), followed by incubation at 4 °C overnight in blocking solution containing 1:5,000 anti-digoxigenin-alkaline phosphatase. After overnight incubation, unbound antibody was removed by 6 × 30 min washings steps at room temperature with TBST 2 (TBST with 0.1% Tween 20 and 0.05% levamisole/tetramisole) and left overnight at 4 °C. At the next day, embryo staining was initiated by 3x 20 min washing steps in alkaline phosphatase buffer (0.02 M NaCl, 0.05 M MgCl2, 0.1% Tween 20, 0.1 M Tris-HCl and 0.05% levamisole/tetramisole in H2O) 3 × 20 min, followed by staining with BM Purple AP Substrate (Roche). At least three embryos were analysed from each mutant genotype. The stained embryos or their limb buds were imaged using a ZEISS SteREO Discovery.V12 with cold light source CL9000 microscope and Leica DFC420 digital camera. The sequences of primers used to generate Triml2, Zfp42, Fat1 are listed in Table S2.

#### LacZ staining in embryos

Whole-mount lacZ reporter staining was performed as previously described with minor adjustments (Lobe et al., 1999). E11.5 mouse embryos were dissected in cold PBS, fixed in 4% paraformaldehyde (PFA) in PBS on ice for 20 min and washed three times in lacZ buffer (2 mM MgCl2, 0.01% sodium deoxycholate, 0.02% Nonidet P-40 in PBS). Embryos were then incubated in staining solution (0.5 mg ml−1 X-gal, 5 mM potassium ferrocyanide, 5 mM potassium ferricyanide in lacZ buffer) at 37°C for a few hours to overnight until desired staining was achieved. Following staining, embryos were washed in lacZ buffer and stored at 4°C in 4% PFA in PBS. Finally, embryos were imaged using a ZEISS SteREO Discovery.V12 with cold light source CL9000 microscope and Leica DFC420 digital camera. LacZ signal was scored in at least 4 replicate embryos and was performed independently by at least two annotators blinded to genotype.

#### qRT-PCR

Hindlimb buds from somite staged E12.5 embryos were dissected, snap-frozen and stored at −80 °C until further processing. Following RNA isolation, cDNA was generated and LacZ mRNA levels quantified by qPCR for at least 3 biological replicates with each in technical triplicate. 2-ΔΔCt method has been used for analysis of relative lacZ expression levels. qPCR primers used: qPCR_LacZ_F, 5′-TTCAACATCAGCCGCTACAG-3′; qPCR_LacZ_R, 5′-CGTCGATATTCAGCCATGTG -3′; qPCR_mGAPDH_F, 5′-TCAAGAAGGTGGTGAAGCAG-3′ and qPCR_mGAPDH_R 5′-ACCACCCTGTTGCTGTAGCC-3′.

#### RNA-seq

Isolated ESCs were trypsinized, heavily feeder depleted, centrifuged and snap frozen. E11.5 forelimb buds were microdissected from wildtype and mutant embryos in cold PBS and immediately snap-frozen for storage at −80°C. Total RNAs were extracted using the RNeasy Mini Kit according to the manufacturer’s instructions. Samples were poly-A enriched, prepared into libraries using the Kapa HyperPrep Kit, and sequenced on a Novaseq2 with 75 bp or 100 bp paired-end reads. RNA-seq experiments were performed at least in duplicates.

#### Sample collection for DamID-seq, ChIP-seq, ATAC-seq, cHi-C and FISH

ESCs were trypsinized, heavily feeder depleted and pelleted by centrifugation. Chicken, opossum and mouse limb buds were microdissected from embryos in cold PBS. Isolated limbs were then trypsinised 5 minutes at 37°C with continuous agitation with a P1000 pipette until no visible clumps remained. Limb cell suspensions were then passed through a 40 μm filter, centrifuged at 250 g for 5 min. Supernatants were then removed from isolated ESCs or limb cells which could then be used for downstream applications.

#### DamID-seq

##### Lentiviral preparation and treatment

DamID was performed as described previously (Robson and Schirmer, 2016). Briefly, lentiviruses encoding the Dam methylase alone (pLgw V5-EcoDam) or fused to lamin B1 (pLgw-EcoDam-V5-Lamin) were generated in 293FT cells. Here, ∼6 million 293FT cells were transfected with 2.8 μg pMD2.G, 4.6 μg psPAX2, and 7.5 μg of pLgw V5-EcoDam or pLgw-EcoDam-V5-Lamin with 36 μl lipofectamine 2000 in 3 ml Optimem. After 16 h, 293FT media was replaced. Virus-containing supernatants were subsequently aspirated after 48 h and 72 h. Viral supernatants were then cleared of cellular debris by 10 min centrifugation at 3,500 rpm and subsequent filtration through a 0.45 μm^2^ low protein-binding PES syringe filter. Viral supernatants were finally purified using the Lent-X concentrator as per manufacturer's instructions and resuspended in Optimem. If not used immediately, aliquots were frozen at -80°C.

To perform DamID, ESCs and cultured E11.5 limb cells were transduced with DamID lentiviruses and harvested 72 or 48 h later, respectively. Specifically, 1,5x10^5^ ESCs were plated feeder-free onto gelatinized 6 well 1 h prior to transduction with DamID lentiviruses. Transduction was then performed overnight after which virus-containing media was removed and cells were plated with feeders in 6 cm plates. After 48 h, contaminating feeders were removed by further feeder-depletion and pure ESCs were isolated by centrifugation. By contrast, isolated E11.5 limb bud cells were directly plated and transduced after 1 h. Virus-containing media was removed 24 h later after which cells were isolated after an additional 48 h.

##### DamID library processing

DamID sample processing was then performed as described previously (Robson and Schirmer, 2016). Briefly, DNA was extracted from cells using the DNeasy tissue lysis kit as per manufacturer’s instructions. 2.5 μg of extracted DNA was then digested by *DpnI* and, following heat inactivation of *DpnI*, was ligated to the DamID adaptor duplex (dsAdR) generated from the oligonucleotides AdRt (5’-CTAATACGACTCACATAGGGCAGCGTGGTCGCGGCCGA-GGA-3’) and AdRb (5’-TCCTCGGCCG-3’) after which DNA was further digested by *DpnII*. To amplify DNA sequences methylated by the Dam methylase, 5 μl of *DpnII* digested material was then subjected to PCR in the supplied buffer in the presence of the 1.25 μM Adr-PCR primer (5’-GGTCGCGGCCGAGGATC-3’), 0.2 mM dNTPs and 1X of the Advantage cDNA polymerase. PCR was performed as previously described after which amplified DNA was purified, processed into NGS libraries using the KAPA HyperPrep kit and analyzed for quality by Bioanalyzer analysis. standard protocols. DamID-seq samples were sequenced 75 or 100 bp paired-end reads and each experiment was performed in duplicates for sequencing.

#### ATAC-seq

ATAC-seq was performed as described previously (Buenrostro et al., 2015). Briefly, 1x10^5^ isolated E11.5 limb cells were employed per biological replicate. Cells were washed in cold PBS, lysed in fresh lysis buffer (10mM TrisCl pH7.4, 10mM NaCl, 3mM MgCl2, 0.1% (v/v) Igepal CA-630) for 2 min on ice, and finally pelleted for 10 min at 500 x g and 4°C. Following supernatant aspiration, nuclei-containing pellets were subjected to transposition using Tn5 Transposase for 30 min at 37° C. Resulting DNA was then purified using MinElute Reaction Clean up kit, eluted in 11 μl of elution buffer and stored in -20° C, if not immediately processed further. Barcoded adapters were added to the transposed fragments by PCR. To avoid saturation in our PCR, we initially performed 5 cycles and extracted a 5 μl aliquot for qPCR to identify the number of cycles required without overamplification. Nextera qPCR primers were used for the amplification. The remaining 45 μl of the PCR reaction were then amplified for the desired number of cycles which never exceeded 12. Finally, samples were purified on AMPure XP beads and eluted in 20 μl. Concentration was measured with Qubit and the quality of the samples was estimated by Bioanalyzer analysis. ATAC-seq samples were sequenced yielding for 50 million 75 bp paired-end reads and each experiment was performed in duplicate.

#### ChIP-seq

ChIP-seq was performed using the iDeal ChIP-seq kit for histones with several modifications. Briefly, ESCs were fixed in 1% paraformaldehyde (PFA)/10% FCS/PBS for 10 min with rotation at room temperature. Fixation was stopped by glycine after which cells were pelleted by centrifugation (8 min, 250 x g, 4°C). Cells were lysed in Lysis buffer (50 mM Tris, pH 7.5; 150 mM NaCl; 5 mM EDTA; 0.5% NP-40; 1.15% Triton X-100; protease inhibitors) for 10 min on ice. Nuclei were resuspended in sonication buffer (10 mM Tris–HCl, pH 8.0; 100 mM NaCl; 1 mM EDTA; 0.5 mM EGTA; 0,1% Na-deoxycholate; 0.5% N-lauroylsarcosine; protease inhibitors). Chromatin was sheared using a Bioruptor until reaching a fragment size of 200–500 base pairs. Afterwards, samples were processed with the iDeal ChIP-seq kit according to the manufacturer’s instructions. For each Histone ChIP 5 μg chromatin was used in combination with antibodies against H3K4me1 (1 μg) H3K4me3 (1 μg), H3K27ac (1 μg) and H3K27me3 (1 μg). Libraries were prepared for sequencing using the KAPA HyperPrep kit and their quality confirmed by Bioanalyzer analysis. ChIP-seq libraries were finally sequenced at 100 bp paired-end reads with all samples analyzed in biological duplicates.

#### ChIPmentation

For chicken embryonic limb buds, ChIPmentation libraries were prepared as previously described (Schmidl et al., 2015). Briefly, dissociated limb cells were filtered through a 70 μm MACS® SmartStrainer before fixation with 1% MeOH-free formaldehyde in PBS on ice for 10 minutes. Fixation was quenched using glycine, and the pellet was collected after centrifugation (3000rpm, 5 min, 4°C. Cells were then lysed in lysis buffer (10mM Tris pH 8.0, 100mM NaCl, 1mM EDTA pH 8.0, 0.5mM EGTA, 0.1% Sodium deoxycholate, 0.5% N-lauroylsarcosine) on ice, before shearing with a Covaris E220 for a fragment distribution of 200-700bp. Sheared chromatin was incubated with appropriate histone antibodies overnight at 4C. Antibody-bound chromatin was immunoprecipitated with Dynabeads™ Protein G. Tn5-mediated ”tagmentation” of pull-downed chromatin was incubated at 37°C for 5min. Chromatin was de-crosslinked with Proteinase K at 65°C overnight. DNA was then purified using the MinElute Reaction Cleanup kit.

Nextera indexing primers (single-indexed) were added during library amplification. The number of PCR cycles for each library was estimated using Ct values as determined by qPCR (where number of cycles = rounded up Ct value +1). After amplification, DNA was cleaned up with AmPure XP beads, and then checked on a TapeStation D5000 HS for size distribution. Size selection was then carried out accordingly, with either a left-sided selection or a double-sided selection. The concentration of final eluted DNA was measured using Qubit HS and checked again on a TapeStation D5000HS. All libraries were sequenced on a Novaseq2 using 100bp paired-end reads. The same histone antibodies used for traditional ChIP-seq were also used here for ChIPmentation.

#### WGBS

Genomic DNA was extracted from ESCs and E11.5 limb buds using the PureLink Genomic DNA Mini Kit following manufacturer’s instructions. gDNA was then sheared in Covaris micro TUBE AFA Fiber Pre-Slit Snap-Cap tubes. Next, the sheared gDNA was purified with the Zymo DNA Clean & Concentrator according to manufacturer’s instructions. Purified DNA was then bisulfite converted using the EZ DNA Methylation-Gold Kit, and WGBS libraries were processed using the Accel-NGS Methyl-seq DNA library kit following manufacturer’s recommendations for each. Libraries were prepared and cleaned using Agencourt AMPure XP beads. The absence of adapters from the final libraries was verified using the Agilent TapeStation. WGBS libraries were sequenced on the NovaSeq6000 yielding 150 base pair paired-end reads.

#### Bisulfite-cloning sequencing

Genomic DNA from E11.5 forelimbs was obtained using Quick-DNA/RNA Microprep Plus Kit. Bisulfite conversion was performed on 1μg of DNA using the EpiTect Bisulfite Kit. Bisulfite-treated DNA was PCR amplified by nested PCR at the Zfp42 promoter and subsequently cloned into a pbluescript vector and sequenced. 10-20 clones from 2 replicates per samples were Sanger sequenced and a total of 12 CpG were analysed with BiQ Analyzer software (Bock et al., 2005).

#### Capture Hi-C (cHi-C)

SureSelect design: The cHi-C SureSelect library was designed over the genomic interval (mm10, chr8: 39022300-48000000) using the SureDesign tool from Agilent.

Fixation: Disassociated ESCs and limb cells were transferred to a 50-ml falcon tube and complemented with 10% FCS/PBS. 37% formaldehyde was added to a final concentration of 2% and cells were fixed for 10 min at room temperature. Crosslinking was quenched by adding glycine (final concentration; 125 mM). Fixed cells were washed twice with cold PBS and lysed using fresh lysis buffer (10 mM Tris, pH 7.5, 10 mM NaCl, 5 mM MgCl2, 0.1 mM EGTA with protease inhibitor) to isolate nuclei. Cell lysis was assessed microscopically after 10-min incubation in ice. Nuclei were centrifuged for 5 min at 480g, washed once with PBS and snap frozen in liquid N_2_.

cHi-C library preparation and sequencing: 3C libraries were prepared from fixed nuclei as described previously (Kragesteen et al., 2018). Briefly, lysis buffer was removed by centrifugation at 400 g for 5 min at 4 °C, followed by supernatant aspiration, snap-freezing, and pellet storage at − 80 °C. Later, nuclei pellets were thawed on ice, resuspended in 520 μl 1× DpnII buffer, and then incubated with 7.4 μl 20% SDS shaking at 900 rpm. at 37 °C for 1 h. Next, 75 μl 20% Triton X-100 was added and the pellet was left shaking at 900 rpm at 37°C for 1 h. A 15-μl aliquot was taken as a control for undigested chromatin (stored at − 20°C). The chromatin was digested using 40 μl 10 U/μl DpnII buffer shaking at 900 rpm at 37°C for 6 h; 40 μl of DpnII was added and samples were incubated overnight, shaking at 900 rpm. at 37°C. On day three, 20 μl DpnII buffer was added to the samples followed by shaking for an additional 5 h at 900 rpm. at 37 °C. DpnII subsequently was inactivated at 65 °C for 25 min and a 50-μl aliquot was taken to test digestion efficiency (stored at − 20 °C). Next, digested chromatin was diluted in 5.1 ml H2O, 700 μl 10× ligation buffer, 5 μl 30 U/μl T4 DNA ligase and incubated at 16°C for 4 h while rotating. Ligated samples were incubated for a further 30 min at room temperature. Chimeric chromatin products and test aliquots were de-cross-linked overnight by adding 30 μl and 5 μl proteinase K, respectively, and incubated at 65 °C overnight. On the fourth day, 30 μl or 5 μl of 10 mg ml−1 RNase was added to the samples and aliquots, respectively, and incubated for 45 min at 37°C. Next, chromatin was precipitated by adding 1 volume phenol-chloroform to the samples and aliquots, vigorously shaking them, followed by centrifugation at 4,000 rpm at room temperature for 15 min. To precipitate aliquot chromatin, 1 volume 100% ethanol and 0.1 volume 3M NaAc, pH 5.6 was added and the aliquots placed at -80°C for 30 min. DNA was then precipitated by centrifugation at 5,000 rpm. for 45 min at 4°C followed by washing with 70% ethanol, and resuspension in 20 μl with 10 mM Tris-HCl, pH 7.5. To precipitate samples, extracted sample aqueous phases were mixed with 7 ml H2O, 1 ml 3M NaAc, pH 5.6, and 35 ml 100% ethanol. Following incubation at −20°C for at least 3 h, precipitated chromatin was isolated by centrifugation at 5,000 rpm for 45 min at 4 °C. The chromatin pellet was washed with 70% ethanol and further centrifuged at 5,000 rpm for 15 min at 4 °C. Finally, 3C library chromatin pellets were dried at room temperature and resuspended in 10 mM Tris-HCl, pH 7.5. To check the 3C library, 600 ng were loaded on a 1% gel together with the undigested and digested aliquots. The 3C library was then sheared using a Covaris sonicator (duty cycle: 10%; intensity: 5; cycles per burst: 200; time: 6 cycles of 60 s each; set mode: frequency sweeping; temperature: 4–7 °C). Adaptors were added to the sheared DNA and amplified according to the manufacturer’s instructions for Illumina sequencing (Agilent). The library was hybridised to the custom designed SureSelect beads and indexed for sequencing (75–100 bp paired-end) following the manufacturer’s instructions (Agilent).

#### Hi-C

Hi-C libraries were prepared as described in a previously published in situ protocol (Melo et al., 2020; Rao et al., 2014). Briefly, ∼1 million cells were fixed in 2% formaldehyde, lysed, and digested overnight with DpnII enzyme. Digested DNA ends were marked with biotin-14-dATP and ligated overnight using T4 DNA ligase. Formaldehyde crosslinking was reversed by incubation in 5 M NaCl for 2 h at 68°C, followed by ethanol precipitation. A S-Series 220 Covaris was used to shear the DNA to fragments of 300–600 bp for library preparation, and biotin-filled DNA fragments were pulled down using Dynabeads MyOne Streptavidin T1 beads. DNA ends were subsequently repaired using T4 DNA polymerase and the Klenow fragment of DNA polymerase I and phosphorylated with T4 Polynucleotide Kinase NK. DNA was further prepared for sequencing by ligating adaptors to DNA fragments, using the NEBNext Multiplex Oligos for Illumina kit. Indexes were added via PCR amplification (4–8 cycles) using the NEBNext Ultra II Q5 Master Mix. PCR purification and size selection were carried out using Agencourt AMPure XP beads. Libraries were sequenced on a NovaSeq2 platform yielding 100 or 150 bp paired-end reads. For each sample, the Hi-C library was created by pooling a total of four technical replicates generated from two different cell isolations cultures in order to ensure higher complexity of the sequencing library.

#### Oligopaint fluorescence *in situ* hybridization with 3D-SIM imaging

Oligopaint library assembly: Oligopaint libraries were constructed as described previously (Beliveau et al., 2015); see the Oligopaints website (https://oligopaints.hms.harvard.edu) for further details. Libraries were ordered from CustomArray in the 92K Oligo pool format. The mm10 coordinates, size, number, density of oligonucleotides and primers used for the libraries are listed in Table S3. Oligopaint oligos were identified using the mm10 ‘balance’ BED files, which consist of 35–41-mer genomic sequences throughout the regions of interest (Beliveau et al., 2018). BED files can be retrieved from the Oligopaints website. Each library contains a universal primer pair followed by a specific primer pair hooked to genomic sequences (119-125 mer oligonucleotides). Oligopaint libraries were produced by emulsion PCR amplification from oligonucleotide pools followed by a ‘two-step PCR’ procedure and the lambda exonuclease method described by Beliveau et al. (2015). The two-step PCR leads to the addition of a specific binding sequence for signal amplification with a secondary oligonucleotide (Sec1-Alexa 488 for green probes or Sec6-Atto 565 for red probes) containing two additional fluorophores. Consequently, each probe carries three fluorophores in total. This strategy allows for the 2-color imaging between different combinations of the oligopaint probes. All oligonucleotides used for Oligopaint production were purchased from Integrated DNA Technologies. Oligonucleotide primer sequences (5′→3′) used for this approach are listed in Table S3.

##### BAC probe preparation

The BAC probe corresponding to the *Fat1* gene was labeled with the Alexa Fluor 555 using the FISH Tag DNA Kit.

##### FISH and immunostaining

FISH was performed as described previously (Szabo et al., 2020). Briefly, 1,5-2 x10^5^ isolated ESCs or E11.5 limb cells were plated from single-cell suspensions onto 0.01% poly-lysine coated coverslips (170 ± 5 μm) for 2 h. Cells were fixed for 10 min in PBS/4% PFA, washed three times in PBS, incubated for 10 min in PBS/0.5% Triton X-100, washed three times in PBS, incubated for 10 min in 0.1 M of HCl and washed twice in 2× SSC/0.1% Tween 20 (2× SSCT). Cells were then incubated in 50% formamide/2× SSCT (20 min at room temperature followed by 20 min at 60 °C). Hybridisation solution was made with 20 μl of FISH hybridisation buffer (50% formamide, 10% dextran sulfate, 2× SSC and salmon sperm DNA (final concentration 0.5 mg/ml)), 0.8 μl of RNase A (10 mg/ml) and Oligopaint probes (primary and secondary probes at 1–3 μM final concentration). When required, co-hybridization of Oligopaints with the *Fat1* BAC probe was performed using 25 ng of BAC probe together with a 50x excess of mouse Cot-1 DNA. Hybridisation solution was deposited on coverslips that were then sealed on glass slides with rubber cement. Slides were placed on a heating block immersed in a water bath for 3 min at 80 °C for denaturation. Probe hybridisation was performed overnight at 42 °C in a dark and humid chamber. Coverslips were removed from glass slides and washed for 15 min in 2× SSCT at 60 °C, 10 min in 2× SSCT at room temperature, 10 min in 0.2× SSC and in PBS. Cells were then washed in PBS/0.1% Tween 20 (PBT) and incubated for 1 h in PBT/2%BSA. Primary antibody (ant-lamin B1, 1:1,000 dilution in PBT/2% BSA) incubation was performed overnight at 4 °C between coverslips and glass slides in a humid and dark chamber. Cells were washed four times in PBT and secondary antibody (anti-rabbit-IgG-Atto 647, 1:100 dilution in PBT/2% BSA) incubation was performed for 1 h at room temperature between coverslips and glass slides in a dark and humid chamber. Last, cells were washed in PBT, stained with DAPI (final concentration at 1 μg/ml in PBS) and washed at least 3 times for 5 min each in PBS. Coverslips were mounted on slides with VECTASHIELD and sealed with nail polish.

##### Image acquisition

3D-SIM imaging was carried out with a DeltaVision OMX V4 microscope equipped with an ×100/1.4 numerical aperture (NA) Plan Super Apochromat oil immersion objective (Olympus) and electron-multiplying charge-coupled device (Evolve 512B; Photometrics) camera for a pixel size of 80 nm. Diode lasers at 405, 488, 561 and 647 nm were used with the standard corresponding emission filters. *Z*-stacks (*z*-step of 125 nm) were acquired using 5 phases and 3 angles per image plane. Raw images were reconstructed using SoftWorx v.6.5 (GE Healthcare Systems) using channel-specific optical transfer functions (pixel size of reconstructed images = 40 nm). TetraSpeck beads (200 nm) (T7280, Thermo Fisher Scientific) were used to calibrate alignment parameters between the different channels. The quality of reconstructed images was assessed using the SIMcheck plugin of ImageJ v.1.52i (Ball et al., 2015).

### Quantification and statistical analysis

#### RNA-seq differential expression analysis

Single-end, 100 bp reads from Illumina sequencing were mapped to the reference genome (mm10) using the STAR mapper (splice junctions based on RefSeq; options: --alignIntronMin20 --alignIntronMax500000 --outFilterMismatchNmax 10). Differential gene expression was ascertained using the DESeq2 package (Love et al., 2014). The cut-off for significantly altered gene expression was an adjusted P value of 0.05.

#### Single cell RNA-seq

The expression of Triml2, *Zfp42*, and *Fat1* genes was investigated in three sc-RNAseq datasets of early mammal development, whole placenta (Marsh and Blelloch, 2020), whole embryo gastrulation (Pijuan-Sala et al., 2019), and whole embryo organogenesis (Cao et al., 2019). For visualization, we used the originally reported Uniform Manifold Approximation and Projection (UMAP) embeddings for the whole placenta and the gastrulation datasets and the t-Distributed Stochastic Neighbor Embedding (tSNE) for the organogenesis dataset. Likewise, we used the reported cell type definitions for visualization. For the whole placenta dataset, we used the “integrated_snn_res.0.6” cell variable to color cell types. UMI counts for *Triml2*, *Zfp42*, and *Fat1* were plotted for all datasets in the range 0 to >2.

#### DamID-seq analysis

Raw reads from DamID-seq experiments were mapped to the mouse mm10 reference genome using the alignment tool BWA-MEM (v.0.7.12) (Li and Durbin, 2009). The counts of mapped reads overlapping a DpnII (GATC) restriction fragment side were normalized by reads per kilobase, divided by the length of the fragment, per million mapped reads (RPKM). Based on these normalized counts the log_2_ fold change between the Dam–Lamin B1 transduced samples and the respective Dam-only-encoding samples was calculated. Finally, LADs were called within 20 kb bins using HMMt which quantifies DamID signal using a modified Baum-Welch algorithm with t emissions (https://github.com/gui11aume/HMMt).

#### ATAC-seq analysis

Raw sequencing fastq files were processed using cutadapt (Martin, 2011) for adapter trimming, Bowtie2 {Langmead, 2012 #2898) for mapping, SAMtools (Li et al., 2009) for filtering, sorting and removing duplicates, and deepTools (Ramírez et al., 2016) for generating coverage tracks.

#### ChIP-seq analysis

Raw sequencing fastq files were processed using STAR (Dobin et al., 2013) for mapping, SAMtools (Li et al., 2009) for filtering, sorting and removing duplicates, and deepTools (Ramírez et al., 2016) for generating coverage tracks.

#### Enhancer prediction

Enhancers were predicted using a series of established tools for ATAC-seq peak prediction and enhancer / promoter prediction. First, Genrich (not published, https://github.com/jsh58/Genrich/) was used to predict ATAC-seq peaks. We filtered for those that overlap a enhancer predicted by CRUP (Ramisch et al., 2019) and do not overlap an annotated TSS (UCSC) or a promoter predicted by eHMM (Zehnder et al., 2019).

#### Enhancer conservation analysis

ATAC-seq peaks and predicted enhancers were projected between mouse, opossum and chicken using a published stepped pairwise sequence alignment approach across multiple bridging species (Baranasic et al., 2021). For a genomic region with conserved synteny, any non-alignable coordinate can be approximately projected from one genome to another by interpolating its relative position between two alignable anchor points. The accuracy of such interpolations correlates with the distance to an anchor point. Therefore, projections between species with large evolutionary distances tend to be inaccurate due to a low anchor point density. Including so-called bridging species increases the anchor point density and thus improves projection accuracy. The optimal choice and combination of bridging species may vary from one genomic location to another. This presents a shortest path problem in a graph where every node is a species and the weighted edges between nodes correspond to a scoring function that represents the distances of genomic locations to their anchor points (|x - a|). The scoring function exponentially decreases with increasing distances |x - a|. The shortest path problem is solved using Dijkstra’s Shortest Path Algorithm (Dijkstra, 1959). The sets of bridging species used here are described in Table S6.

Projected elements from ATAC-seq peaks were then classified into directly (DC), indirectly (IC) or not conserved (NC) according to the following criteria: DC elements overlap a direct sequence alignment between the reference and the target species. IC elements do not overlap a direct alignment, but are projected with a score > 0.99, i.e. either overlapping or in direct vicinity to a multi-species anchor. A score of > 0.99 means that the sum of the distances from the element and its intermediate projections to their respective anchor points is < 150 bp throughout the optimal bridging species path. The remaining peaks are classified as non-conserved (NC).

#### cHi-C and Hi-C analysis

##### cHi-C analysis

Raw fastq files had read lengths of 75 bp and 100 bp, respectively. In a preprocessing step, fastq files with 100 bp read length were trimmed to 75 bp to achieve comparable initial read lengths for all samples. Afterwards, fastq files were processed with the HiCUP pipeline v0.8.1 (no size selection, Nofill: 1, Format: Sanger) for mapping, filtering and deduplication steps (Wingett et al., 2015). The pipeline was set up with Bowtie 2.4.2 for mapping short reads to reference genome mm10 (Langmead and Salzberg, 2012). If replicates were available, they were merged after the processing with the HiCUP pipeline. Binned and KR normalized cHi-C maps (Knight and Ruiz, 2013; Rao et al., 2014) were generated using Juicer tools v1.19.02 (Durand et al., 2016). Only read pairs for region chr8:39,030,001-48,000,000 and with MAPQ≥30 were considered for the generation of cHi-C maps.

In addition to the original cHi-C maps, custom reference genomes were derived from mm10 for the ΔD1+2 deletion line. cHi-C and Hi-C maps were displayed as linear-scaled heatmaps in which very high values were truncated to improve the visualization.

##### Hi-C analysis

Fastq files were processed with the Juicer pipeline v1.5.6 (Durand et al., 2016) (CPU version) using bwa v0.7.17 (Li and Durbin, 2010) for mapping short reads to the reference genomes mm10 (mouse), hg19 (human), galGal6 (chicken), monDom5 (opossum), susScr11.1 (pig), and AmexG_v6.0-DD (axolotl), respectively. Replicates were merged after the mapping, filtering and deduplication steps of the Juicer pipeline. Juicer tools v1.7.5 (Durand et al., 2016) were used to generate binned and KR normalized Hi-C maps from read pairs with MAPQ≥30.

For compartment analysis, hic-files were converted at 100kb bin size to the cool format using hic2cool (v0.8.2) (https://github.com/4dn-dcic/hic2cool) and balanced using cooler (v0.8.5) (Abdennur and Mirny, 2020). Afterwards, compartment analysis was performed using cooltools (v0.3.0) (https://github.com/open2c/cooltools) and using the GC content as reference track.

TADs were identified by applying TopDom v.0.0.228 on 50-kb binned and KR-normalized maps using a window size of 10 (Shin et al., 2016). Insulation scores were calculated using Cooltools (https://github.com/open2c/cooltools/tree/v0.4.1)

#### Gene co-regulation in TADs analysis

To calculate gene-expression correlations, we downloaded FANTOM stage 5’ CAGE TPM data (https://fantom.gsc.riken.jp/5/data/). We discarded samples annotated as belonging to ‘reference’ ‘whole body’ or similar samples, and also excluded testis and related tissues from the analysis. We also removed all libraries with fewer than 1 million reads, and all peaks with less than 32 reads across all samples. Overlapping each peak with the Gencode M23 annotation, we assigned peaks to genes if they overlapped a Gencode exon for that gene, or were less than 200bp upstream of a TSS. Peaks not overlapping a gene were discarded, and the counts for all of a gene’s peaks were summed.

Since the FANTOM data contained the resulting gene x sample count matrix was then normalized as per (Alam et al., 2020) – normalized counts-per-million for each sample. As many of the sample in the FANTOM CAGE data were highly correlated (due e.g. to being replicates or adjacent time points), we performed hierarchical clustering on the 829 remaining datasets, and then merged libraries with a pearson correlation of 0.95 or greater, resulting in a final 349 metasamples. Co-expression between two genes was then defined as pearson correlation across these 349 metasamples.

To identify housekeeping genes (Figure S6B), we replicated the procedure used by FANTOM previously (Consortium et al., 2014). Here, the 2D density of median and maximum normalized expression over all samples is first plotted, and then setting a cutoff on median expression that separated ubiquitous from non-ubiquitous genes. To assess the relationship between co-expression and linear gene distance separation or TAD co-occupancy and co-expression we next identified TADs in ESCs, E11.5 limb buds and cortical neurons (Bonev et al., 2017; Kraft et al., 2019). Plotting co-expression as a function of distance revealed, as expected, a strong relationship between linear proximity in the genome and co-expression. Since genes sharing TADs are necessarily more likely to be closely spaced, we plotted (log_10_) linear distance against co-expression separately for pairs either sharing or not sharing a TAD, pooling gene pairs with similar linear distance in a moving average over 2000 points. Mean Corr. Values were calculated by averaging correlations for all gene pairs within a TAD (Figure 6C).

#### WGBS processing

Raw reads were subjected to adapter and quality trimming using cutadapt (version 2.4; parameters: --quality-cutoff 20 --overlap 5 --minimum-length 25; Illumina TruSeq adapter clipped from both reads), followed by trimming of 10 nucleotides from the 5’ end of the first read, 15 nucleotides from the 5’ end of the second read and 5 nucleotides from the 3’ end of both reads (Kechin et al., 2017). The trimmed reads were aligned to the mouse genome (mm10) using BSMAP (version 2.90; parameters: -v 0.1 -s 16 -q 20 -w 100 -S 1 -u -R) (Xi and Li, 2009). Duplicates were removed using the ‘MarkDuplicates’ command from GATK (version 4.1.4.1; --VALIDATION_STRINGENCY=LENIENT --REMOVE_DUPLICATES=true) (McKenna et al., 2010). Methylation rates were called using mcall from the MOABS package (version 1.3.2; default parameters) (Sun et al., 2014). All analyses were restricted to autosomes and only CpGs covered by at least 10 reads and at most 150 reads were considered for downstream analyses.

#### Differentially methylated region (DMR) calling

DMRs were called using metilene (version 0.2-8; parameters: -m 10 -d 0.2 -c 1 -f 1) (Jühling et al., 2016) using two replicates per condition and filtered for a Q-value < 0.05. DMRs were assigned to overlap a promoter if 20% of the DMR or 20% of the promoter overlapped using bedtools ‘intersect’ (Quinlan and Hall, 2010). Genes on the X, Y or M chromosome were not considered for the analysis as these chromosomes were omitted from the methylation analysis. Promoters with and without DMRs were then subsequently assigned to the TAD with the maximum overlap.

#### SBS-polymer modelling with NE-attachment

We simulated the 3D structure of the *Fat1/Zfp42* locus in ESC and E11.5 limb buds using a Strings and Binders Switch (SBS) polymer model that incorporates NE-attachment as described below (Barbieri et al., 2012; Chiariello et al., 2016; Nicodemi and Prisco, 2009).

##### Polymer model

Briefly, the SBS polymer model simulates a chromatin filament as a string with N beads, possessing potential binding sites for specific interacting molecules (binders). The binder concentration c and bead-binder interaction energies $E_{int}$ control the system’s state through a coil-globule transition occurring when they are above a threshold (Barbieri et al., 2012; Chiariello et al., 2016). The type and location of binding sites specific for different regions of the *Zfp42/Fat1* locus were inferred from ESC or E11.5 limb cHi-C data using PRISMR (mm10 chr8: 40300000 - 46200000; 20 Kb resolution) (Bianco et al., 2018). This machine-learning based algorithm returns the minimal arrangement of binding sites to fit the input. As output, the best polymer modelling the *Fat1/Zfp42* locus was generated with 13 distinct types of binding sites in each condition. From these polymers, we obtain a set of 3D structures representing chromatin conformations in ESC and E11.5 limb through standard Molecular Dynamics simulations (see below).

##### Details of Molecular Dynamics simulations

In order to build an ensemble of 3D structures representing the *Fat1*/*Zfp42* locus in E11.5 limb and ESC cell lines, we perform extensive Molecular Dynamics (MD) simulations (Chiariello et al., 2016). For simplicity, bead and binders have the same diameter σ=1 and mass m=1 in dimensionless units. A standard truncated Lennard-Jones (LJ) potential models the hard-core repulsion between the objects. By contrast, interaction between beads and binders is modelled with an attractive LJ potential with distance cutoff ranging from $R_{int}=1.3\sigma$ to $R_{int}=1.5\sigma$ and an interaction intensity, given by the minimum of the LJ potential, within the range of $E_{int}=3.1-8.2K_{B}T$. An additional non-specific, weaker interaction (in the $E_{int}=2-3K_{B}T$ range) is set among binders and the polymer. Consecutive beads of the polymer are linked by FENE bonds (Kremer and Grest, 1990) with standard parameters (length $R_{0}=1.6\sigma$ and spring constant $K_{FENE}=30K_{B}T/\sigma^{2}$). Beads and binders move through Brownian dynamics according to the standard Langevin equation (Allen and Tildesley, 2017) with temperature T=1, a friction coefficient ζ=0.5 and an integration time step Δt=0.012 (dimensionless units). The polymer is first initialized as a Self-Avoiding-Walk and the binders are randomly located in the simulation box, then the system is equilibrated up to approximately 10^8^ timesteps. From each model, we perform up to 10^2^ independent simulations in which polymer configurations are sampled every 5^∗^10^5^ timestep once equilibrium is reached. Simulations are performed with the LAMMPS package (Plimpton, 1995).

##### Modelling the nuclear envelope

To model the NE, we introduce a spherical wall of radius R within the simulation box. Polymer beads can attractively interact with NE though a short range, truncated LJ potential with affinity $E_{NE}$ ranging from $0.0K_{B}T$ to $10K_{B}T$ and cutoff distance $r_{cutoff}=2.5\sigma$. Among the NE-bead interaction energies tested, the structures obtained immediately after the NE-polymer adsorption (around $1.2K_{B}T$) generated structural measurements that most closely matched those observed by FISH (Figure S4). Alternatively, beads interact with NE only through a purely repulsive LJ potential. The NE sphere radius is set to R=40σ. In order to define the interaction state (repulsive or attractive) of each polymer bead with NE, we employ DamID data for each wild or mutant ESC/limb sample. Briefly, we compute the average DamID signal in each 20kb window and evaluate its sign. Polymer beads associated with an average positive DamID signal are classified as attractively interacting with NE. Conversely, beads associated with a negative signal experience only a repulsive interaction. In this way, regions enriched with DamID tend to attach to the NE in the model. In our simulations, the NE is introduced after the SBS (polymer+binders) system is equilibrated, as described in the previous section. Then, in order to ensure the complete interaction of the polymer with the NE, the system is equilibrated up to other 7^∗^10^7^ timesteps.

##### Quantification of measurements

Pairwise distance distributions are extracted from the population of 3D polymer structures as previously described (Chiariello et al., 2016; Conte et al., 2020). For each pair of objects, we first compute the center of mass of the polymer beads belonging to that object, then we evaluate the distance between the centers of mass. This distance is then averaged over the last 20 frames of each simulation. In order to map dimensionless length scale into physical units we compare pairwise distances measured by FISH. In total, we compare six different probe pairs (D1-D2, Fl1-Fl2, *Zfp42*R-D1, *Zfp42*R-D2, *Zfp42*R-Fl1, *Zfp42*R-Fl2) both in E11.5 limb and ESCs, for each pair we equalize the model and experimental median and then average over the different probe pairs. The resulting length scale mapping factor is σ=44nm.

Distances from NE shown in Figures S4E and S4F are estimated by computing: $d_{NE}=R-\left|{\vec{r}}_{CM}-{\vec{r}}_{NE}\right|$, where R is the model NE radius, ${\vec{r}}_{CM}$ is the position of the center of mass of the object and ${\vec{r}}_{NE}$ is the position of the NE center. Physical distances are then obtained using the mapping factor σ previously calculated from the comparison with pairwise FISH distances.

Pairwise overlaps between two objects shown in Figure S4I are obtained by using the following expression: $overlap_{12}=A_{12}/\left(A_{1}+A_{2}-A_{12}\right)$, where $A_{1}$ and $A_{2}$ are the surfaces of 2D projections associated to object 1 and object 2 respectively and $A_{12}$ is their common area. For simplicity, 2D projections are approximated as circles whose radii $R_{1}$ and $R_{2}$ are estimated as gyration radii from the projected coordinates, so $A_{1}=\pi R_{1}^{2}$ and $A_{2}=\pi R_{2}^{2}$. In this way, overlapping areas can be easily estimated using standard geometric relations. Indeed, given the distance d between the centers of the projected objects and supposing, without loss generality, $R_{2}>R_{1}$, we have a partial overlap if $R_{2}-R_{1}<d<R_{1}+R_{2}$. In this case: $A_{12}=R_{2}^{2}\alpha_{1}-d_{1}\sqrt{\left(R_{2}^{2}-d_{1}^{2}\right)}+R_{1}^{2}\alpha_{2}-d_{2}\sqrt{\left(R_{1}^{2}-d_{2}^{2}\right)}$, where $d_{1}=(R_{2}^{2}-R_{1}^{2}+d^{2})/2d$ and $\alpha_{1}=arccos\left(d_{1}/R_{2}\right)\;$ (analogous relations hold for $d_{2}$ and $\alpha_{2}$). If $d\geq R_{1}+R_{2}$, we impose $A_{12}=0$, i.e. objects are well separated in space; finally, if $d\leq R_{2}-R_{1}$, we set $A_{12}=\pi R_{1}^{2}$, i.e. object 1 is completely contained within object 2. Three body overlaps shown in Figures S4E and S4G involving *Zfp42*R or Fat1 with D1+D2, are defined as: $overlap_{123}=\left(A_{12}+A_{13}\right)/\left(A_{1}+A_{2}+A_{3}-A_{12}-A_{13}-A_{23}\right)$, where object 1 can be *Zfp42*R or Fat1. As for 3D distances, overlap values are averaged over the last 20 frames of each simulation. Analogously, a geometric mapping factor of 1.2 is found when comparing with pairwise experimental medians.

Sphericity is defined using the standard formula: $sphericity=(\pi^{1/3}{\left(6V\right)}^{2/3})/A$, where A and V are area and volume of the object respectively. Area and volume are estimated from the coordinates of the polymer beads belonging to the region under consideration by means of a 3D convex hull approximation, computed with the Python package scipy.spatial. Sphericity measurements can be viewed in Figures S4E and S4H.

Contact maps are computed as previously described (Chiariello et al., 2016; Conte et al., 2020). We first measure the distance $r_{ij}$ between any two beads i and j. If the distance is lower than threshold (7.5σ in Figures S4B and S4C), the beads are in contact. For each considered condition (without NE and with NE at different interaction energies), aggregated matrices are obtained over the different independent simulations. Visual and quantitative comparisons reveal a general good agreement between model and cHi-C data in both cell lines (Pearson r=0.90 and distance-corrected (Bianco et al., 2018) Pearson $r^{\prime }=0.72$ in HL, r=0.91 and $r^{\prime }=0.64$ in ESC, genomic distances >100kb). Subtraction matrices D are defined as the simple bin-wise difference $D_{ij}=x_{ij}^{NE}-x_{ij}$, where $x_{ij}^{NE}$ and $x_{ij}$ are the entries of the contact maps with and without NE respectively.

##### Polymer graphics

Polymer 3D snapshots shown in Figures 3 and S4 are representative single molecule structures taken from real MD simulations. Regions corresponding to Fl1, D1, *Zfp42*R, D2, Fat1, Fl2 are differently colored. A slice of the simulated NE is rendered as a thick spherical wall colored as in FISH imaging. To clarify the relationship between the polymer and NE, each image is presented from the same point-of-view through a geometrically calibrated 3D rotation matrix. For visual purposes, polymers are shown in a coarse-grained version of a smooth third-order polynomial spline passing through bead coordinates.

See Table S5 for a summary of statistical measurements from polymer modelling.

#### Oligopaint FISH image analyses

Image analysis was performed using Fiji and MATLAB (R2018-2019 and image processing toolbox). For *overlap intermingling fraction* and *combined sphericity* measurements, z-stacks of regions of interest (ROIs) of 3×3 μm^2^ surrounding FISH signals were extracted and smoothed using a 3D Gaussian filter (sigma = 0.5 pixel). FISH channels were then segmented in 3D using automatic Otsu’s method. Only ROIs containing 1 FISH segmented object per channel (or at least 1 object for the combined D1+D2 FISH) larger than 0.04 μm^3^ were kept for further analyses. *Object intermingling fraction* of *Zfp42R* or *Fat1* with D1+D2 (Figures 3D and S4) was obtained by dividing the overlapping volume between *Zfp42R* or *Fat1* and D1+D2 by the volume of *Zfp42R* or *Fat1*. *Overlap* (Figures 3D and S4) correspond to the Jaccard Index between the two segmented FISH objects. For *combined sphericity* calculation, FISH segmented objects from the two channels were merged into one, and only ROIs containing 1 merged object were considered for the analysis. *Combined sphericity* was defined as $=\;\left(\pi^{\frac{1}{3}}{\left(6V\right)}^{\frac{2}{3}}/A\right)$ where V is the volume of the segmented object and A its surface area. For distance to lamin analysis, z-stacks of ROIs surrounding individual nuclei were extracted and smoothed using a 3D Gaussian filter (sigma = 0.5 pixel). FISH channels were segmented using a threshold value corresponding to 20% of the maximum pixel intensity. For a given FISH channel, only nuclei containing 2 segmented FISH objects larger than 0.04 μm^3^ were kept for further analysis. For each FISH object, an ROI surrounding its maximum and minimum z-coordinates was extracted and the lamin channel was segmented using Otsu’s method. Lamin segmented objects smaller than 0.02 μm^3^ were discarded and Lamin segmented channel was processed using the MATLAB imfill function. 3D Euclidean distance transform of the segmented Lamin channel was calculated using the MATLAB bwdistsc function and distance to the centroid of the FISH segmented object was extracted.

See Table S5 for a summary of statistical measurements from FISH analyses.

## Data Availability

• All data reported in this paper will be shared by the lead contact upon request. This paper analyzes existing, publicly available data whose accession numbers are listed in the key resources table. Sequencing data generated in this study are available at the NCBI Gene Expression Omnibus, GEO: GSE185775. • This paper does not report original code. • Any additional information required to reanalyze the data reported in this work paper is available from the lead contact upon request.

## Supplemental information

Table S1. List of mutant ESCs with sgRNAs, donor knockin plasmids and genotyping primers indicated, related to Figures 1, 2, 3, 4, 5, S1, S3, S4, and S5 Breakpoint genotyping primers are used to detect deletions (del), site specific integration (ssi) or specific KI-construct (kic).

Table S2. List of oligonucleotides for DamID processing, promoter cloning and genotyping, related to Figures 1, 2, 3, 4, 5, S1, S3, S4, and S5

Table S3. Summary of Oligopaint library with regions of interest indicated and oligonucleotide primer sequences, related to Figures 3, 4, and S4

Table S4. Summary of statistics for RNA-seq, qPCR, related to Figures 2, 5, S1, and S3

Table S5. Summary of statistics for FISH and polymer modelling analyses, related to Figures 3 and S4

Table S6. Summary of species used evolutionary comparison, related to Figures 1 and S1
